# Beyond the Average: Trends in Extreme Sodium Intake in the U.S. Population, 2003–2018

**DOI:** 10.3390/nu17121975

**Published:** 2025-06-11

**Authors:** Yutong Chen, Jingyan Wang, Kristin E. Leonberg, Kenneth Kwan Ho Chui, Lynne M. Ausman, Elena N. Naumova

**Affiliations:** 1 Friedman School of Nutrition Science and Policy, Tufts University, Boston, MA 02111, USA; yutong.chen@tufts.edu (Y.C.); jingyan.wang@tufts.edu (J.W.); kristin.leonberg@tufts.edu (K.E.L.); kenneth.chui@tufts.edu (K.K.H.C.); lynne.ausman@tufts.edu (L.M.A.); 2 School of Medicine, Tufts University, Boston, MA 02111, USA

**Keywords:** sodium intake, dietary sodium, nutrient intake trends, temporal trend analysis, cardiovascular diseases

## Abstract

Background: Excessive sodium consumption is a major contributor to hypertension and cardiovascular disease (CVD), yet most nutrition surveillance systems focus primarily on average intake measurements, neglecting patterns of extreme sodium consumption. Public health strategies need to consider how high sodium intake patterns evolve across the entire population over time. Objective: The study analyzed sodium consumption patterns across US populations through NHANES data from 2003 to 2018 while focusing on percentiles and extreme consumption levels. The study evaluated the accelerations and decelerations of sodium consumption while analyzing its trends in vulnerable groups (people with self-reported hypertension, CVD, heart attack, and stroke) along with chronological time and age changes. Methods: The analysis of NHANES data involved 60,663 participants between ages 5–80 years from eight survey cycles (2003–2018). The research used non-linear Gaussian regression to model sodium intake (mg/day) with age and the NHANES cycle treated as continuous predictor variables. We classified intake curves into four main intake patterns, increase with acceleration (IA), increase with deceleration (ID), decrease with acceleration (DA), and decrease with deceleration (DD), and estimated turning points to reflect critical risk periods in 16 selected subgroups defined by age, sex, and self-reported health conditions. We also examined temporal trends in intake extremes through individual-level and population-level data. Results: In most adult subgroups, we observed a non-linear pattern over time, indicating that sodium intake initially increased and then plateaued or declined around the turning point of ~20–30 years. Only girls demonstrated a steady decline in sodium intake over time, while in boys, we detected an alarming accelerating increase. The intake at upper percentiles remained extremely high: approximately 10% of the population consumed more than 5000 mg of sodium per day, which is more than twice the recommended limit of 2300 mg/day. Participants with CVD, heart attack, and stroke had a lower average intake than those without, but intake remained above recommended levels. Conclusions: From 2003 to 2018, sodium intake in the U.S. showed no signs of meaningful decline, especially at the upper extreme. These findings suggest that current efforts are insufficient to meet the WHO’s global target of a 30% sodium reduction by 2030.

## 1. Introduction

Cardiovascular disease (CVD) is the leading cause of death in the world, contributing to about 17.9 million deaths every year and 32% of all deaths, according to the World Health Organization’s 2019 report [1]. Hypertension, a major risk factor for CVD, affects an estimated 1.39 billion adults globally, with nearly half unaware of their condition [2,3]. Although the prevalence of hypertension is lower in high-income countries compared to low- and middle-income countries, it still affects approximately 349 million people [2,3]. In the United States, CVD affects an estimated 127.9 million adults, and nearly 47% of adults have hypertension, according to the report published by the American Heart Association in 2024 [4]. While stroke and myocardial infarction continue to be major contributors to CVD-related morbidity and mortality—with one person experiencing a heart attack every 40 s—recent data suggest concerning trends [4]. After decades of decline, CVD mortality rates have begun to rise again, partly driven by worsening risk factors such as diabetes, obesity, and population aging [4].

The excessive intake of sodium is a significant preventable and modifiable factor that leads to high blood pressure and, thus, to cardiovascular disease [5,6]. The INTERSALT study, which involved 52 populations in 32 countries, has identified sodium intake as a positive predictor of systolic blood pressure [5]. Indeed, the populations with lower sodium intake had lower mean blood pressure and less age-related rises in blood pressure [5,6]. More recent evidence has confirmed that high salt intake remains one of the top three dietary risk factors worldwide and that population-wide sodium reduction lowers blood pressure and reduces the burden of CVD, all-cause mortality, kidney disease, stomach cancer, and osteoporosis [7]. Randomized trials have demonstrated that salt reduction lowers blood pressure among both hypertensive and normotensive individuals, even when added to antihypertensive treatments [7]. As a result, salt reduction programs are now recognized as cost-effective public health strategies. In line with this evidence, the World Health Organization (WHO) has set a global target to reduce population sodium intake by 30% by 2030 to help prevent millions of deaths from stroke and heart disease [8]. The WHO has also developed the SHAKE technical package to guide countries in implementing salt reduction strategies [9].

However, there are still many populations with high levels of sodium intake despite well-established efforts in public health [8,10,11]. The major dietary sources of sodium on a global level are bread and other baked products, processed meats, dairy products, cereals, packaged snacks, and especially ultra-processed foods [12,13,14]. The consumption of sodium has been of concern in the United States for several decades. The 2020–2025 Dietary Guidelines for Americans (DGA) suggest that sodium intake should be less than 2300 mg per day for adults, yet most people exceed this amount [15]. The Scientific Report of the 2025 Dietary Guidelines Advisory Committee pointed out that sodium intake is elevated among all age groups and emphasized that the main sources of sodium in the American diet are processed and restaurant foods. These structural dietary patterns make it challenging for people to meet the recommended sodium levels through personal dietary choices alone [15].

Importantly, results from the Trials of Hypertension Prevention (TOHP) follow-up study, which used repeated 24 h urine collections, reported a 17% increase in cardiovascular event risk for every additional 1000 mg of daily sodium intake [16]. This underscores the urgent need for public health interventions to reduce sodium consumption. One promising strategy is the Dietary Approaches to Stop Hypertension (DASH) diet, which emphasizes fruits, vegetables, and low-fat dairy products while limiting sodium and has been shown to significantly lower blood pressure [17]. However, despite the available evidence and recommendations on sodium intake, the level is still very high in the United States [18,19]. More than 70% of daily sodium intake comes from processed and restaurant foods, which leaves little room for people to decrease their sodium intake without changes in the food system [14]. Therefore, it is important to know the trends of sodium intake over time, especially among people at high risk of CVD.

The aim of this study is to examine sodium intake records among US populations using NHANES data from 2003 to 2018, focusing on intake distribution, specifically the extremes, rather than relying only on mean intake. By analyzing the entire distribution of sodium intake and percentile trends, we aim to identify population subgroups at higher risk and evaluate how the sodium intake pattern changes over the years. Specifically, we aim to quantify potential acceleration (or deceleration) of sodium intake in two temporal components: chronological time and age. This analysis was conducted across eight cycles in 16 selected groups, including individuals with self-reported specific medical conditions, including hypertension, CVD, heart attack, and stroke. In particular, by examining acceleration and deceleration patterns over time, we can identify whether sodium intake is declining quickly enough to meet population-level targets. A slowing or plateau in trends may indicate that current interventions are insufficient enough, especially among high-risk population groups. We expect there has been a significant reduction in sodium intake in the last few years, especially in high-risk groups, such as those with hypertension and CVD, due to sodium reduction efforts. First, we examined the temporal trend in mean sodium intake covering the age between 5 and 80 years, using individual-level data at single-year age resolution. Next, we examined the acceleration (or deceleration) in temporal trends in the extremes of sodium intake. Finally, we discussed implications for public health and policy making by considering both the central tendencies and the extremes in the sodium intake.

## 2. Data and Methods

### 2.1. Dietary Sodium Assessment

This study uses data from the National Health and Nutrition Examination Survey (NHANES), conducted by the Centers for Disease Control and Prevention (CDC) [20]. The NHANES is a cross-sectional survey of a nationally representative sample of the U.S. population [21]. It collects data on dietary intake, health status, and health service utilization through structured interviews, physical examinations, and laboratory assessments [21]. The present analysis is based on dietary sodium intake data collected from eight sequential NHANES cycles, spanning from 2003 to 2004 to 2017–2018. NHANES cycles are coded from 0 to 7, corresponding to survey years 2003–2004 (cycle 0), 2005–2006 (cycle 1), 2007–2008 (cycle 2), 2009–2010 (cycle 3), 2011–2012 (cycle 4), 2013–2014 (cycle 5), 2015–2016 (cycle 6), and 2017–2018 (cycle 7).

In the NHANES database, dietary sodium intake was assessed using 24 h dietary recall methods conducted by trained interviewers, following the USDA Automated Multiple-Pass Method (AMPM), which is a commonly used approach for collecting typical daily dietary intake data [22,23,24]. Starting from the 2003–2004 survey cycle, two 24 h recalls were collected for each participant: the first (Day 1) was administered in person at the Mobile Examination Center (MEC), while the second (Day 2) was conducted via telephone 3 to 10 days later [25,26]. The AMPM involves a structured sequence. First, participants were asked to list all foods and beverages consumed on the day prior to the interview. This was followed by a probe for commonly forgotten items, such as snacks or condiments [23,24,26]. Interviewers then collected detailed information about the time of consumption, food preparation methods, and portion sizes [23,24,26]. The interview concludes with a final review to ensure completeness [23,24,26]. Daily total sodium intake in mg/day was calculated by the NHANES (CDC) based on the reported food and beverage intakes for both days. However, only Day 1 data were used in this study due to lower completion rates on Day 2 and concerns about potential underreporting during telephone-based interviews, the method of data collection for Day 2 dietary recall [27,28]. Although using both days could theoretically reduce within-person variation, we used only Day 1 data because of their higher completion rate, consistent in-person administration, and reduced risk of underreporting due to interviewer method differences [27,28]. This approach ensures more consistent and comparable intake estimates across the full sample.

For children aged 12–17 years, the dietary interview was conducted independently. For children aged 6–11 years, interviews were proxy-assisted, and for those aged 5 years, a proxy respondent (typically a parent or caregiver familiar with the child’s intake) provided all dietary information. Written parental consent was obtained for all participants aged 5–17 years [25]. In addition, assent was obtained from children and adolescents aged 7–17 years, following NHANES protocols for ethical participation of minors in federally conducted surveys [25].

### 2.2. Sample Selection and Exclusions

The final analytical sample consisted of 60,663 participants drawn from eight NHANES cycles spanning 2003–2004 to 2017–2018. Data from 80,213 participants from all cycles were first merged into a single dataset. Exclusions were applied for missing Day 1 dietary recall data for 3305 participants, invalid dietary responses as flagged by the NHANES for 6288 participants, and age under 5 years for 10,056 participants. After exclusions, the sample included 18,194 children aged 5–17 years (9198 boys and 8996 girls) and 42,469 adults aged 18 years and older (20,637 males and 21,832 females). We did not apply further exclusion thresholds for high sodium intake values beyond those already flagged as invalid by the NHANES. While some participants’ consumptions exceeded 20,000 mg/day, we retained them to reflect the full range of reported behaviors, recognizing that such extreme intake levels are uncommon but can still occur in real-world eating patterns.

### 2.3. Self-Reported Health Conditions Records

In addition to the dietary recall data, self-reported health conditions were assessed through standardized interview questions administered during the NHANES household interview [26]. Adult participants were asked whether they had ever been told by a doctor or other health professional that they had specific medical conditions, including hypertension, CVD, heart attack, and stroke. For hypertension, participants were asked, “Have you ever been told by a doctor or other health professional that you had hypertension, also called high blood pressure?” [29]. CVD status was determined based on responses to a similar question, “Has a doctor or other health professional ever told you that you had coronary heart disease?” [29]. Similarly, heart attack and stoke were assessed using the same format of questions, with the condition name substituted accordingly, including “heart attack (also called myocardial infarction)” or “stroke.” For each question, response options included: Yes, No, Refused, or Don’t Know [29].

In this study, these self-reported responses were recoded into binary indicators to classify participants into paired subgroups (with vs. without each condition), where “with” includes those who answered “Yes”, and “without” includes those who answered “No”, “Refused”, or “Don’t Know”. Additionally, we created a composite indicator variable for the presence of any condition. Among the 42,469 adult participants, subgroup sizes were as follows: 14,374 (33.8%) were classified as having hypertension and 28,095 (66.2%) without; 1677 (3.9%) with heart disease and 40,792 (96.1%) without; 1753 (4.1%) with heart attack and 40,716 (95.9%) without; and 1566 (3.7%) with stroke and 40,913 (96.3%) without.

An indicator variable for having any condition was created to reflect the presence of one or more conditions. Participants were classified as having at least one condition if they responded “Yes” to at least one of the four questions (N = 15,356), and as having no condition if they answered “No”, “Refused”, or “Don’t Know” to all four (N = 27,113). There were 11 adults who were missing responses to all four health condition questions, and 2743 adults were missing data for at least one condition. However, missing health condition data were not used as exclusion criteria.

### 2.4. Statistical Analysis

To examine trends in extreme sodium intake values, we estimated several intake percentiles for each NHANES cycle across 16 selected groups—organized as 8 paired comparisons: all children vs. all adults; boys vs. male adults; girls vs. female adults; adults with vs. without each of the four self-reported health conditions (hypertension, heart disease, heart attack, and stroke); and adults with no reported condition vs. those with at least one condition.

To explore and communicate patterns in sodium intake across the NHANES cycles, we used multiple data visualization techniques tailored to both distributional and temporal analyses. Scatter plots of mean age-specific intake for each cycle, with superimposed curves smoothed with the LOWESS method [30], were used to visualize age-specific sodium intake trends in the whole sample stratified by sex. LOWESS smoothing allowed for flexible, non-parametric modeling of age–intake relationships without assuming a specific functional form.

For each cycle and group, we calculated the mean, standard deviation, minimum, maximum, and seven selected percentiles (P5, P10, P25, P50 (median), P75, P90, and P95) to capture both central tendency and variability. We also computed skewness and kurtosis coefficients to better understand the shape and dispersion of intake distributions. We also produced cumulative distribution plots and kernel density estimate (KDE) plots to summarize the distribution and spread of sodium intake. Cumulative plots were stratified by cycle and sex, illustrating the proportion of the population consuming less than specific intake thresholds over time. In contrast, KDE plots combined data across all NHANES cycles to visualize the overall shape and variability of the intake distribution, stratified by sex and overlaid with key percentiles (P5–P95) for reference. This descriptive analysis provided the foundation for subsequent trend analyses and helped identify shifts in sodium intake distributions over time and across population subgroups.

To compare mean sodium intake between paired subgroups, including adults vs. children, males vs. boys, females vs. girls, and participants with vs. without health conditions, we conducted independent two-sample *t*-tests. These tests were conducted based on individual-level sodium intake data and evaluated whether group differences were statistically significant. Due to unequal group sizes and variances, Welch’s *t*-test was applied. All tests were two-sided, with a significance level at 0.05.

### 2.5. Modeling Sodium Intake with Individual Level Data

First, we examined the temporal trend in mean sodium intake in select groups across eight cycles over a 16-year period, covering the age range from 5 to 80 years at single-year age resolution and aiming to detect potential acceleration and deceleration in the temporal components of both chronological time (NHANES cycle) and age (participant age) using individual level data. We assessed trends in average sodium intake by applying Gaussian regression models, with daily dietary sodium intake (mg/day) as the dependent variable and the NHANES cycle (coded from 0 to 7, representing the 2003–2004 and 2017–2018 cycles) and age (treated as a continuous variable) as the primary independent variables, representing chronological time and age, respectively. Unweighted data were used in the regression models to preserve the distributional properties of sodium intake and enable more accurate modeling of non-linear trends and turning points. Since NHANES weights are designed to optimize population means, adding weights would introduce distortions in individual-level extreme intake behavior, which are the central points in our analysis.

Four sequential models were developed to explore both linear and non-linear trend components for time and age. Model 1 included only a linear term for the cycle. Model 2 added a quadratic term for the cycle to capture potential non-linear acceleration or deceleration in intake trends. Model 3 included an additional variable for participant age, and Model 4 added a quadratic term for age to account for possible curvilinear age effects. These four models were applied to each of the 16 selected groups:

Model 1: $Sodium\;Intake=\beta_{0}+\beta_{1}Cycle$

Model 2: $Sodium\;Intake=\beta_{0}+\beta_{1}Cycle+\beta_{2}{Cycle}^{2}$

Model 3: $Sodium\;Intake=\beta_{0}+\beta_{1}Cycle+\beta_{2}{Cycle}^{2}+\beta_{3}Age$

Model 4: $Sodium\;Intake=\beta_{0}+\beta_{1}Cycle+\beta_{2}{Cycle}^{2}+\beta_{3}Age+\beta_{4}{Age}^{2}$

For these models, regression coefficients (*β*) were estimated, tabulated, and used to produce trend interpretations. The combinations of signs and magnitudes of the regression parameters (*β*_1_, *β*_2_, *β*_3_, and *β*_4_) derived from Model 4 form distinct local behaviors and overall patterns for temporal or age-related trends. To provide an interpretation of these combinations, we use a generic model, y = b_0_ + b_1_*x* + b_2_*x*^2^, where *x* > 0 and corresponds to age or cycle in Model 4. Table 1 outlines interpretations of different combinations of signs and magnitudes of b_1_ and b_2_ (corresponding to *β*_1_, *β*_3_ and *β*_2_, *β*_4_ in Model 4, respectively).

The table lists conditions when linear or quadratic terms dominate and provides an estimate of the inflection point as a ratio of b_1_ and b_2_ when the dominance switches from one term to another at *x* = |b_1_|/|b_2_|. The combination of signs of b_1_ and b_2_ coefficients and the magnitudes indicate the *local behavior*, or a trend near an intercept b_0_ (in Model 4, *x* = 0 represents the first cycle; note that for the age-related term, the minimum age was 5 in children and 18 in adults). We distinguish four local behaviors, coded as an increase with acceleration (IA), increase with deceleration (ID), decrease with deceleration (DD), and decrease with acceleration (DA). In addition to local behaviors, we distinguish *global patterns* like *rise-then-fall, fall-then-rise*, and overall decline based on the position of turning point *x* = −b_1_/2b_2_, which is essential for scenarios with opposite signs. For example, in the case of ID, the overall global pattern would be *rise-then-fall,* with a dominant decline pattern when the range of *x*-values after the turning point is larger than prior to the turning point. These patterns help distinguish whether sodium intake is increasing, decreasing, plateauing, or changing at an accelerating or decelerating rate across cycles or over age spans.

### 2.6. Modeling Sodium Intake with Population Level Data

Next, we examined the temporal trend in sodium intake in selected percentiles of sodium intake (P5, P10, P25, P50 (median), P75, P90, and P95), aiming to detect potential acceleration and deceleration across eight cycles using population-level data focusing on low and high sodium intakes. Model 5 included the NHANES cycle as a linear term, while Model 6 added a quadratic term for the cycle to capture potential shifts and provide insight into both central and extreme intake behaviors:

$$\begin{array}{l} \mathrm{Model}\;5:Y_{p}=\beta_{0}+\beta_{1}Cycle\\ \mathrm{Model}\;6:Y_{p}=\beta_{0}+\beta_{1}Cycle+\beta_{2}{Cycle}^{2} \end{array}$$

where *Y_p_* represent the estimated value of the *p*th percentile of daily sodium intake (mg/day), and *p* corresponds to P5, P10, P25, P50, P75, P90, or P95. We applied these models for each of seven selected percentiles and for each group. To illustrate temporal trends in intake across subgroups, we generated heatmaps showing predicted sodium intake at selected percentiles over time, using outputs from regression Model 6. These visualizations enabled comparisons across age, sex, and health condition status and allowed us to demonstrate shifts in the extremes of intake distributions.

### 2.7. Sensitivity Analysis

To conduct the proposed analysis and apply non-linear regression models to track changes over chronological time and with respect to age at single-year resolution, we must use unweighted data. Thus, unweighted data were used: (a) to produce key visualizations based on individual-level data and preserve the true shape of the distribution and (b) to estimate skewness and kurtosis coefficients. While NHANES sampling weights are essential for generating nationally representative estimates at the population level, they are not designed to reflect individual-level intake distributions [31,32]. To address potential concerns regarding the use of unweighted data, we performed sensitivity analysis to detect the differences in conclusions. We provided population-level estimates (e.g., weighted percentiles per cycle) and modeling estimates for weighted data. The sensitivity analysis allowed us to evaluate the extent of difference introduced by applying survey weights and to ensure transparency in how intake distributions are presented and interpreted.

### 2.8. Programming Software Used

Analyses were conducted in R (version 2024.09.1). Scatter plots, line graphs, and density graphs were created in R using the ggplot2 package. For weighted analyses, statistical models were run in SAS (version 9.4) to account for the NHANES’s complex survey design, and predicted results used in heatmap were exported from SAS and completed in Microsoft Excel.

## 3. Results

**Figure 1:**
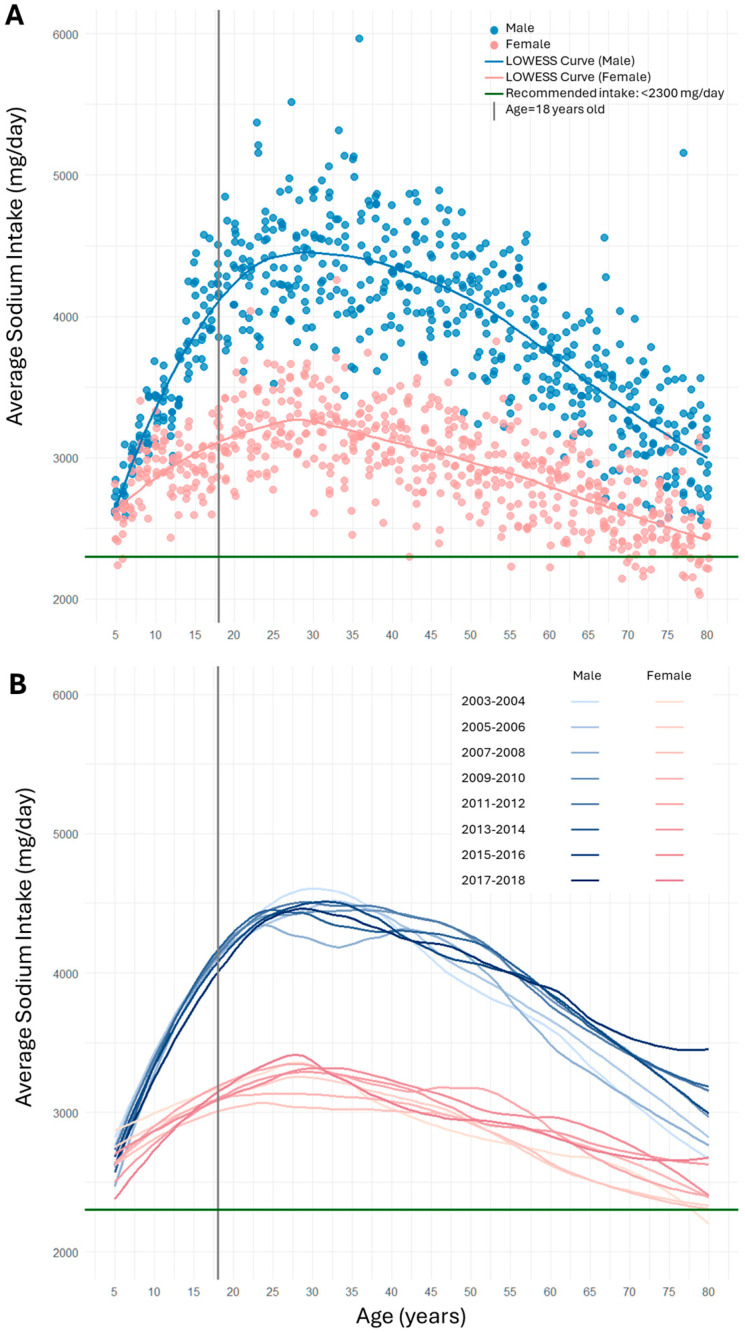
Temporal and age-related patterns in average sodium intake (mg/day) stratified by sex among NHANES participants (2003–2018). Panel (**A**) is a scatter plot of average sodium intake by age, sex, and NHANES cycle, with LOWESS curves fitted across all cycles combined (2003–2018). Each dot represents the mean sodium intake for a specific age–sex group in each cycle. Panel (**B**) depicts LOWESS curves fitted separately for each NHANES cycle (without individual data points), highlighting temporal changes in sodium intake patterns across cycles (2003–2018).

The age-related patterns of sodium intake are presented in Figure 1. Panel A shows a scatter plot of average sodium intake (mg/day) for each year of age and each cycle, with the smoothed LOWESS curves fitted across all combined NHANES cycles from 2003 to 2018, stratified by sex.

Males consistently exhibited higher sodium intake than females across the age range. Children exhibited a sharp increase in sodium intake. Younger adults tended to have higher intake levels compared to older individuals. Sodium intake peaked around age of 25–30, after which it began to decline, forming a ∩-shaped or *rise-then-fall* pattern. Panel B displays separate LOWESS curves for each cycle, allowing visualization of temporal variations across chronological time. Although curves from different survey cycles largely overlapped, suggesting minimal year-to-year differences overall, there is a likely trend among older adults: sodium intake appeared higher in more recent cycles (indicated by a shift from lighter to darker colors in the lower portion of the curves). This pattern was observed in both males and females.

Descriptive statistics of sodium intake in sixteen groups of eight distinct pairs, including five groups representing health condition status, are shown in Table 2. This table summarizes sodium intake, pooled across NHANES cycles from 2003 to 2018, key percentiles (P5 through P95), and skewness and kurtosis coefficients to reveal the distributional extremes and the substantial proportion of population exceeding the recommended limit. 

In nearly all subgroups, sodium intake exceeded the recommended limit of 2300 mg/day beginning as early as the 25th percentile, meaning that at least 75% of individuals consumed more than the recommended level. Median intakes were also way above recommendations, ranging from 2645 mg/day among girls to 3617 mg/day among male adults. Moreover, by the 90th percentile, many groups exceeded 4600 mg/day, which is twice the recommended intake. Skewness values ranged from 1.10 to 1.65 across groups, indicating right-skewed distributions. Kurtosis up to 7.35 suggests heavy tails and a higher frequency of extreme intake. Individuals with listed health conditions consistently consumed less sodium than those without, particularly at higher percentiles, though even in those groups, median values still exceeded 2300 mg/day. The observations were consistent with the patterns observed in Figure 1, where LOWESS curves show higher sodium intake across most age groups for males compared to females. Across all subpopulations, some individuals report a typical-day intake level exceeding 20,000 mg. Detailed descriptive analyses by cycle are provided in Table S1. All *t*-tests comparing sodium intake between subgroups yielded *p*-values < 0.0001, indicating highly statistically significant differences in mean intake.

**Figure 2:**
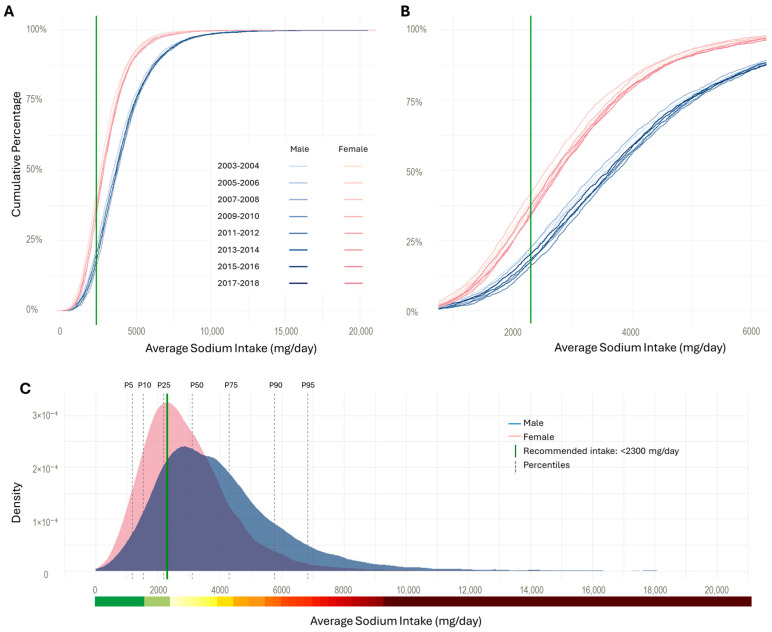
Distributions of sodium intake among U.S. adults (2003–2018). Panel (**A**) presents the cumulative distribution of sodium intake (mg/day), stratified by sex. Panel (**B**) is a zoomed-in view of Panel (**A**), focusing on intake between 1000 and 6000 mg/day. In Panel (**A**,**B**), each curve represents the cumulative proportion of the population at or below a given intake level. The green reference line at 2300 mg/day indicates the recommended intake. Panel (**C**) depicts a kernel density plot of sodium intake (mg/day), stratified by sex. Shaded background regions correspond to percentile zones (P5, P10, P25, P50, P75, P90, and P95).

To further illustrate the sodium intake among U.S. adults, we plotted cumulative distributions, stratified by sex, for each cycle with a zoomed-in view (Figure 2). 

Based on the cumulative distribution, 37% of females and 20% of males consumed sodium at or below the recommended intake level of 2300 mg/day. At the median (P50), sodium intake was approximately 3100 mg/day for males and 2500 mg/day for females. Males also demonstrated greater variability in intake around the median, with a spread of approximately 260 mg compared to 180 mg among females. Panel C presents the overall density curves, stratified by sex, further highlighting the differences in extreme intakes.

**Figure 3:**
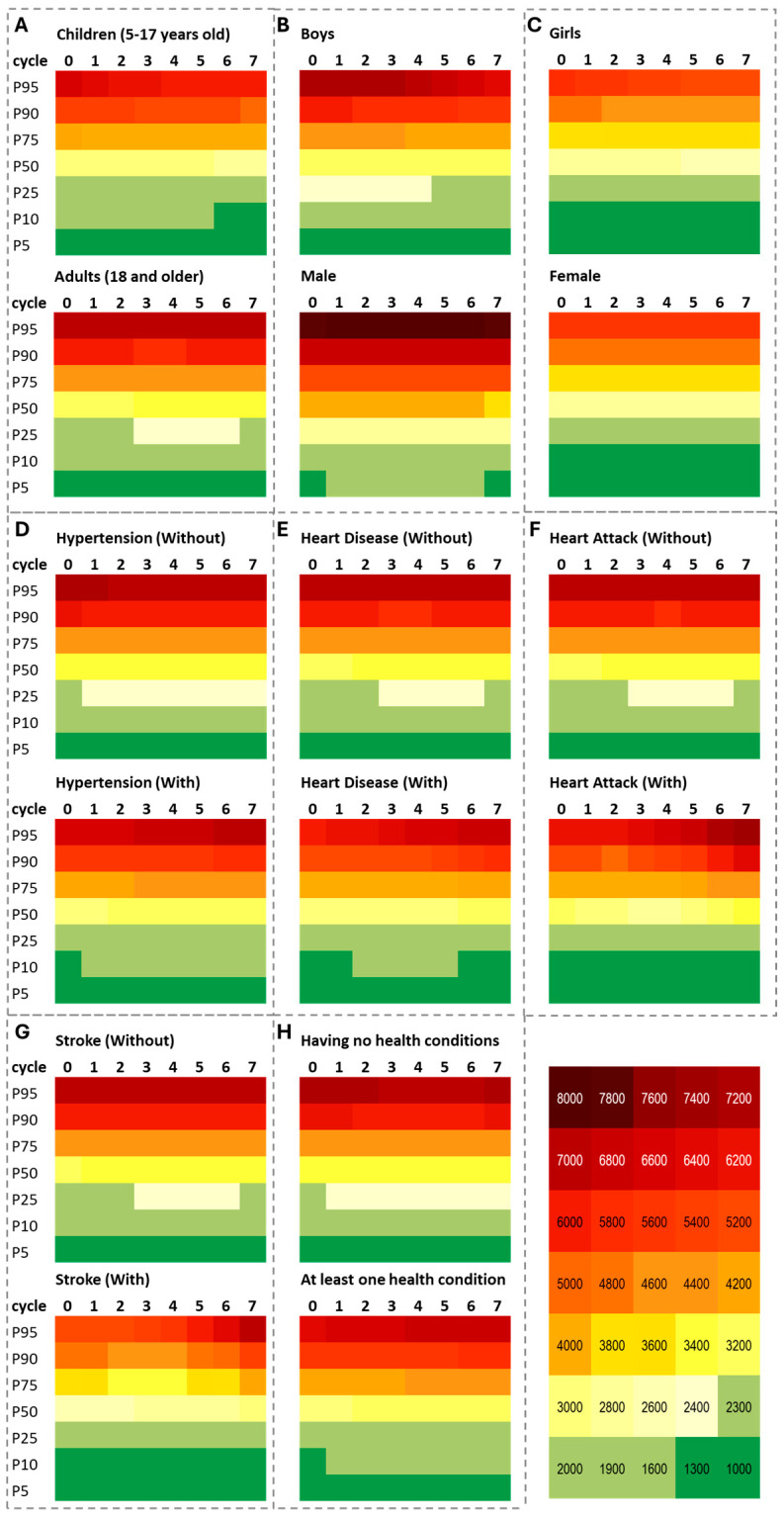
Heatmaps of predicted sodium intake (mg/day) across NHANES cycles by percentile and subgroup. Panels show predicted sodium intake at selected percentiles across NHANES cycles, stratified by key demographic and health condition groups. Panel (**A**)—children aged 5–17 years vs. adults aged 18 and older; Panel (**B**)—boys vs. male adults; Panel (**C**)—girls vs. female adults; and Panels (**D**–**H**)—adults by health condition status, including Panel (**D**)—hypertension, Panel (**E**)—heart disease, Panel (**F**)—heart attack, Panel (**G**)—stroke, and Panel (**H**)—presence of any condition. Sex-specific results are shown in Panels (**B**,**C**). In Panels (**D**–**H**), “Without” indicates participants without the specified condition or with missing responses, while “With” indicates confirmed presence of the condition. NHANES cycles are coded from 0 to 7, corresponding to survey years 2003–2004 (cycle 0), 2005–2006 (cycle 1), 2007–2008 (cycle 2), 2009–2010 (cycle 3), 2011–2012 (cycle 4), 2013–2014 (cycle 5), 2015–2016 (cycle 6), and 2017–2018 (cycle 7).

The results of non-linear regression modeling (Model 4) are summarized in Table 3 and Figure 3. Across all 16 examined groups, sodium intake generally exhibited a curvilinear relationship with age, characterized by increasing intake until 22–26 years of age, followed by a decline in later years. The observed ∩-pattern of sodium intake increase followed by plateau (for children) or a decline (for adults) was consistent among all studied groups as well as adults with and without health conditions. In contrast, the detected age-related increase with acceleration among boys is quite alarming.

Temporal trends in sodium intake showed a wide range of variations. The average intake is likely to decline over time among girls. While adults overall experienced a statistically significant increase in average intake over time, this trend was much less pronounced among adults with self-reported stroke, heart disease, and heart attack. In contrast, participants without chronic conditions or cardiovascular disease tended to show modest increases in sodium intake with some deceleration in recent cycles. Detailed results for Models 1–3 and Model 4 are provided in Table S2 and Table S3, respectively.

Figure 3 illustrates the temporal trends for selected percentiles as predicted by non-linear Model 6 applied to each cycle and each percentile in each group (for detailed modeling results, see Table S4). The heatmaps show the temporal patterns in extremes, with dark red colors indicating high intakes. High extremes are concentrated in panels representing adult males, indicating the greatest intake growth according to regression models. Notably, the 95th percentile among adult males increased significantly across cycles, reaching levels of about 8000 mg/day, which is approximately 3.5 times the recommended intake. This finding reinforces the urgent need for targeted public health strategies to reduce extreme intake. Similarly, participants with self-reported health conditions also showed rising intake at the upper percentiles across cycles, a concerning trend given that these groups are expected to lower their sodium intake. In contrast, females, children, and those without reported health conditions display lower and more stable intake levels, consistent with more modest or no trends (depicted by broader green and yellow areas). The concentration of extremely high intake (≥7000 mg/day) in certain panels supports the observed deceleration in trends over time, as intake levels appear to have plateaued at the higher end of the distribution in recent cycles.

The results of the sensitivity analysis are shown in Tables S5–S7, indicating minimal differences between weighted and unweighted data in terms of the findings for descriptive statistics and individual-level models, with less conservative estimates in population-level models.

## 4. Discussion

In this study, we examined temporal trends in sodium intake among U.S. children and adults using NHANES data from 2003 to 2018, applying both individual-level and population-level distribution-based analyses to detect important shifts over chronological time with age. By leveraging non-linear models to quantify acceleration and deceleration, we evaluated trends across the sodium intake distribution, revealing patterns often invisible to traditional mean-based approaches. We analyzed 16 selected subgroups organized into eight pairs (based on age, sex, and health condition comparisons) and classified their trends using a proposed framework of local behaviors (IA, ID, DA, and DD) and global dominant patterns (*rise-then-fall* or *fall-then-rise*) based on estimated turning points (Table 1 and Table 2).

This approach allowed us to identify potential risk groups, periods, and subtleties of plateauing, accelerating, and decelerating. Importantly, our analysis went beyond the usually reported trends of means; it also examined trends in percentiles and intake extremes, offering a detailed classification of trends across the full intake distribution and across subgroups (Table 3). This granularity enhances our understanding of how intake is shifting within and across populations and provides critical context for evaluating progress toward sodium reduction goals.

For temporal trends (cycle), most subgroups showed an initial increase followed by pronounced deceleration, particularly among adults, males, and those without hypertension, heart disease, heart attack, or stroke. Girls were the only subgroup showing a decrease with deceleration, signaling some progress. Several groups—including children, female adults, and those with heart disease, heart attack, or stroke—showed no temporal trend, underscoring a desirable yet insufficient change to reach the needed reduction in sodium consumption.

The substantial increase in age-related trends revealed an alarming picture. Boys stood out with a unique increase in acceleration patterns, highlighting a troubling trajectory as they move into adulthood and older age. Other adult subgroups, including male adults and those without hypertension or cardiovascular conditions, showed *rise-then-fall* or ID-∩ patterns, indicating that intake increases with age, peaks at the turning point, and then flattens and begins to decline. Among females, the pattern showed a notable overall decline. The turning points calculated from the non-linear models provide important insights into when sodium intake peaks, identifying critical windows for intervention. Many of these turning points occurred in early adulthood (ages 20–30), revealing this life stage as an especially critical period to target for intervention to alter long-term intake trajectories and reduce overall sodium intake.

Our analysis of extreme intake trends further underscores this challenge: the proportion of individuals consuming the highest levels of sodium continues to increase (Figure 3). Across subgroups, many individuals reported daily intakes exceeding 7000 mg, with some surpassing 20,000 mg/day. These heavy consumers are likely to carry the greatest clinical risk and may disproportionately contribute to the national burden of diet-related disease. Without meaningful reductions among these top percentiles, progress toward the 2030 goal is unlikely. Targeting these individuals must become a public health priority, as they likely drive a large portion of the diet-related disease burden. While some reduction in intake with age may reflect physiological changes, such as reduced caloric needs due to muscle mass loss among older adults [33], these natural shifts alone are insufficient to drive the population-wide improvements needed.

In our analysis, the sodium intake among participants with health conditions was substantially and systematically lower than their counterparts across the whole intake distribution, including the extremes. These detected differences might be due to their awareness of the risks of high sodium intake, medication compliance, older age, etc. Despite the strong positive association between dietary sodium intake and the risk of hypertension, the associations of sodium intake with the risk of CVD and chronic kidney diseases are inconsistent [34]. Some studies have found a positive association between dietary sodium intake and these clinical outcomes, whereas others have found inverse, J-shaped, or U-shaped associations [16,35,36,37]. High sodium intake appeared to account for about 10% to 30% of CVD-related deaths among the high risk groups [38]. These conflicting findings can likely be partly explained by methodological limitations, such as systematic and random error in sodium measurements, reverse causality, insufficient statistical power, residual confounding, and inadequate follow-up duration [34,37]. Nevertheless, dietary sodium reduction has been recommended to lower population BP levels and the risk of hypertension.

These observed age and sex differences also raise an important policy implication. Although the age-related decline in sodium intake may partly reflect changes in physical activity, dietary habits, or overall energy requirements [39,40,41,42], it also highlights a broader issue: unlike most vitamins and minerals, which have age- and sex-specific dietary reference intakes [43,44], sodium recommendations remain uniform for all population groups [15]. Yet men generally consume more calories than women, and younger adults tend to have higher energy intake than older adults [45,46,47]. In this study, we focused on absolute intake rather than energy-adjusted intake because our primary goal was to characterize population-level intake distributions and extreme values, which have direct implications for policy and public health guidance. Additionally, we used percentile-based, non-parametric methods that are less affected by energy adjustment. However, the lack of differentiation in current guidelines warrants reconsideration, especially in light of these physiological and behavioral differences.

When considering national policies and individual strategies, the ideal trajectory is not merely a reduction in sodium intake but a decline with acceleration, a steepening downward slope that would be necessary to meet the World Health Organization’s target of reducing sodium intake by 30% by 2030 [8]. Yet, based on our findings, reality falls far short of this benchmark, which was also concluded by a WHO 2023 report [48]. Most subgroups exhibited either increasing trends or minimal deceleration, with little evidence of the sustained, accelerated declines needed. Achieving the WHO goal will require far more aggressive and targeted public health strategies, particularly among high-risk populations.

In addition to individual-level behaviors, structural determinants also play a substantial role in shaping sodium intake patterns across the US population. Numerous studies have shown that lower socioeconomic status is associated with higher sodium consumption, often due to limited access to fresh foods and the predominance of ultra-processed, high-sodium foods in affordable food environments [49,50,51]. For example, a systematic review estimated that individuals in lower-income groups consume approximately 14% more sodium than higher-income groups, which corresponds to 503 mg/day [49]. Furthermore, limited access to healthy food retailers, aggressive marketing of processed foods, and regional limitations to grocery stores all contribute to excess sodium consumption [52,53,54]. Although our analysis did not directly incorporate these factors, future work should examine how these broader contextual influences interact with demographic and health characteristics to affect intake.

Several national and international initiatives have been developed to reduce sodium intake through various guidance and reformulation. The U.S. Food and Drug Administration issued the Draft Guidance for Industry on Voluntary Sodium Reduction Goals, which outlines target sodium concentrations for processed and packaged foods. The WHO’s SHAKE package provides a global policy framework for sodium reduction, including reformulation, labeling, consumer education, surveillance, and environment [9]. However, most countries, including the US, still rely on voluntary policies rather than mandatory ones [48,55]. According to the WHO’s 2023 global report on sodium reduction progress and a midterm systematic review, only small fractions of countries have implemented legally binding sodium limits [48,55]. As a result, global progress remains insufficient to meet the WHO’s 2025 non-communicable disease prevention goal and the 2030 target of a 30% reduction in population sodium intake [48,55,56]. In contrast, tax policies targeting sugar-sweetened beverages (SSBs) have shown strong effectiveness in reducing population-level intake and could be adapted to sodium-rich foods [57,58,59]. Similarly, front-of-package warning labels, standardized sodium labeling, and ongoing national surveillance could help consumers make more informed choices and support policymakers in designing targeted interventions. As voluntary strategies have shown only modest effects, more assertive regulatory policies may be necessary to achieve meaningful reductions among the highest consumers.

Our study presents several strengths, including a large, nationally representative sample, consistent modeling approaches across subgroups, a structured way of assessing acceleration and deceleration patterns, and the use of both individual-level and population-level evaluation. By applying the proposed framework alongside rich NHANES data, we provide findings that are accessible to both researchers and policymakers. What sets our work apart from traditional studies is the integration of three key elements: non-linear modeling, attention to intake extremes, and global pattern classification based on turning point estimation, an approach not many studies are using [60]. This analytical trifecta offers a more detailed and actionable perspective on population sodium patterns by capturing the dynamics of intake changes over time with the simultaneous detection of populations at risk.

Despite the strengths of this study, certain methodological limitations must be acknowledged. The self-reported 24 h dietary recalls in the NHANES introduce systematic measurement errors through underreporting, which nutritional epidemiology recognizes as a fundamental challenge, especially when participants try to report sodium intake [61,62]. A prior study estimated this underreporting at around 452 mg/day [62]. In addition, short-term assessments like these lack the ability to capture the long-term effects of sodium intake on cardiovascular health.

Moreover, an important limitation of this study is the absence of objective sodium biomarkers, such as 24 h urinary sodium excretion, in most NHANES cycles analyzed. This constraint limits our ability to validate self-reported dietary sodium intake and may affect the accuracy of our findings. Beyond these limitations, biological factors such as salt sensitivity may also help explain the variability in sodium-related outcomes across individuals, yet these are rarely captured in epidemiologic studies. Current cohort studies often have brief observation periods, potentially underestimating the cumulative effects of sodium consumption over many years. Our results suggest that uniform sodium recommendations cannot adequately address key differences across age groups or account for biological variation like salt sensitivity and that future guidelines should incorporate both age and salt sensitivity into their frameworks.

In addition to the limitations discussed above, our analysis did not include other important cardiovascular risk factors such as overweight, obesity, or diabetes. These factors are well-established contributors to cardiovascular disease risk and have been extensively studied in relation to sodium intake using the NHANES and other datasets [63,64,65,66]. Many prior studies have focused on examining associations between sodium intake and specific clinical outcomes or risk profiles [63,64,65,67]. In contrast, our study was designed to evaluate trends in the distribution and extremes of sodium intake over time, rather than to model individual-level disease risk. Given that our focus was on population-level intake patterns and their acceleration or deceleration over age and time, it is unlikely that including additional covariates like BMI would substantially alter the observed trajectory patterns. It is possible that with the expansion of GLP-1 medications—glucagon-like peptide-1 receptor agonists, a class of drugs used to treat type 2 diabetes and, in some cases, moderate-to-severe obesity—we might see a notable change in BMI at the population level with time [68,69]. Nonetheless, future research could integrate broader cardiometabolic risk factors to explore how such factors may interact with sodium consumption trends or modify intake behaviors over time.

Although the distribution of sodium intake is right-skewed and exhibits high kurtosis, we applied non-linear Gaussian modeling with ordinary least-squares (OLS) estimation. This approach allowed us to design the classification system of patterns using model parameters. In our further model improvement research, we will explore the use of distributions that better capture the skewed nature of the intake distribution. While OLS could provide unbiased and consistent estimates of conditional means even under non-normal residuals [70] and the large NHANES sample mitigates concerns about distributional violations, the future model improvements are warranted.

Our sensitivity analysis showed negligible differences between weighted and unweighted modeling results, lending confidence to the robustness of our estimates. The existing statistical tools for applying weights are still limited and restrict the use of novel approaches to examine individual-level data. To further assess the robustness of our findings, it could be of use to explore a wide temporal range to enable more accurate trend forecasting that incorporates demographic changes, medication compliance, improvements in therapeutic approaches to treat population at risk, and health policies that support healthy lifestyles.

The present analysis was limited to eight cycles of data from the NHANES to ensure consistent survey methodology, nutrient databases, and variable coding. Earlier cycles used different methods to collect dietary data and processing and documentation procedures, which could create compatibility issues when studying time trends at the granular level. Previous studies, including CDC analyses of sodium intake trends from 2003 to 2010 and 2003 to 2016, highlighted persistently high levels of sodium consumption in the US population despite public health recommendations [39,71]. One study stitched together mean sodium intakes from NHANES data from 1999 to 2016 among adults in subgroups based on age, sex, race and ethnicity, and sodium-sensitive chronic diseases and derived similar conclusions [72]. We offered a more nuanced analysis that emphasizes trend acceleration to reinforce previous findings, while providing updated insights into intake patterns and subgroup-specific trends. Although the NHANES recently released a newly available cycle (August 2021–August 2023) [73], we excluded it from this analysis to maintain methodological comparability with earlier data. Specifically, the newly released cycle differs substantially in several aspects: it did not include oversampling by race and Hispanic origin, both 24 h dietary recalls were conducted via telephone, and response rates were lower than in pre-pandemic cycles [74]. Future research should incorporate these new data to evaluate sodium intake trends during and after the COVID-19 pandemic. The extension of the study period that covers a quarter of century would allow researchers to detect both generational patterns and cohort effects and assess sodium-related health risks that occur throughout life.

## 5. Conclusions

Despite decades of public health initiatives, sodium consumption in the United States remains well above World Health Organization (WHO) targets, especially among those with extreme intakes. Achieving the 2030 sodium reduction goals seems unlikely without substantial changes in consumer behavior, food industry practices, and regulatory policies. Our findings suggest that universal strategies may be insufficient to shift intake patterns at the population level and that tailored, targeted interventions are likely needed to reduce intake among the highest-consuming groups. This study provides a comprehensive assessment of sodium intake trends among U.S. children and adults from 2003 to 2018, revealing persistently high intake and limited progress in most subgroups, particularly at the upper percentiles. These patterns highlight the need for targeted interventions to reduce intake among high-consuming groups, slow the alarming rise in sodium intake in boys, and support the steady decline among receptive groups. As the 2030 WHO sodium reduction goals approach, coordinated efforts across individuals, the food industry, and policymakers will be critical to achieving meaningful change. To support long-term improvements, nutrition surveillance should be integrated into broader systems for monitoring dietary quality and nutrition-related risk factors [75,76]. This approach can help guide more comprehensive, data-driven public health strategies.

## Figures and Tables

**Table 1:** A structured approach to define local trends and global patterns based on the signs and relative magnitudes of regression b_1_ and b_2_ estimated form the regression model y = b_0_ + b_1_*x* + b_2_*x*^2^ and the turning point is *x* = −b_1_/2b_2_.

Condition	Sign	Interpretation: Local Behavior and Global Pattern
The quadratic term dominates over time: b_2_*x*^2^ > b_1_*x* for large *x*, or equivalently *x* > b_1_/b_2_	b_1_ > 0 (increasing linear trend) b_2_ > 0 (positive curvature)	IA—increase with acceleration (fast incline): the slope increases as *x* increases, resulting in a steepening upward trend over the observed range.
The linear term dominates for low values of *x*: b_1_*x* > ∣b_2_∣*x*^2^ or *x* ≤ b_1_/∣b_2_∣	b_1_ > 0 (increasing linear trend) b_2_ < 0 (negative curvature)	ID—increase with deceleration (slow incline): the slope increases but at a decreasing rate as *x* increases; The *Rise-then-fall* patterns: this may appear as a *rise-then-fall* (∩-shaped) curve if a turning point exists within the observed *x*-range, or as a gradually flattening upward trend, otherwise.
The linear term dominates for low values of *x*: ∣b_1_∣*x* > b_2_*x*^2^, or *x* ≤ ∣b_1_∣/b_2_	b_1_ < 0 (decreasing linear trend) b_2_ > 0 (positive curvature)	DD—decrease with deceleration (slow decline): the slope decreases as *x* increases, but the rate of decline slows down; The *fall-then-rise* patterns: this may appear as a *fall-then-rise* (∪ shape) if a turning point exists within the observed range, or as a flattening downward trend.
The quadratic term reinforces the decline: ∣b_2_∣*x*^2^ > ∣b_1_∣*x*, or *x* > ∣b_1_∣/∣b_2_∣	b_1_ < 0 (decreasing linear trend) b_2_ < 0 (negative curvature)	DA—decrease with acceleration (fast decline): the slope decreases as *x* increases, resulting in a downward trend over the observed range.

**Table 2:** Descriptive summary statistics, percentiles and extremes, and distribution shape parameters of sodium intake among children (ages 5–17) and adults (ages 18 and older) stratified by sex and in adults based on health conditions, pooled across eight NHANES cycles (2003–2018).

	Statistics ^a^				Percentiles and Extremes ^b^									Shape ^c^	
Group	N	Mean	SD	Range	Min	P05	P10	P25	P50	P75	P90	P95	Max	Skew	Kurt
Children (5–17)	18,194	3157 *	1601	20,325	0	1190	1496	2085	2861	3887	5143	6135	20,325	1.62	5.50
Adults (18 and older)	42,469	3442	1849	25,949	0	1172	1521	2177	3104	4300	5757	6833	25,949	1.64	5.87
Boys	9198	3438 *	1749	20,325	0	1318	1633	2257	3100	4219	5644	6734	20,325	1.60	5.22
Male adults	20,637	3962	2046	25,949	0	1397	1804	2549	3617	4940	6537	7753	25,949	1.47	4.70
Girls	8996	2870 *	1375	15,976	0	1110	1390	1940	2645	3527	4580	5397	15,976	1.42	4.33
Female adults	21,832	2950	1482	21,004	0	1052	1360	1936	2713	3688	4758	5618	21,004	1.62	7.35
Hypertension (Without) ^d^	28,095	3550	1899	25,949	0	1207	1574	2255	3206	4417	5929	7048	25,949	1.64	5.92
Hypertension (With)	14,374	3230 *	1728	20,999	5	1123	1440	2045	2899	4047	5377	6433	21,004	1.60	5.48
Heart disease (Without)	40,792	3457	1860	25,949	0	1176	1524	2187	3116	4318	5785	6867	25,949	1.64	5.88
Heart disease (With)	1677	3060 *	1504	11,474	5	1096	1412	2004	2814	3846	4990	5948	11,479	1.10	1.98
Heart attack (Without)	40,716	3459	1854	25,949	0	1182	1532	2192	3117	4319	5782	6866	25,949	1.64	5.87
Heart attack (With)	1753	3039 *	1675	15,720	5	984	1280	1893	2723	3819	5118	6090	15,725	1.65	5.79
Stroke (Without)	40,913	3463	1856	25,949	0	1185	1533	2193	3124	4321	5788	6864	25,949	1.64	5.90
Stroke (With)	1556	2873 *	1551	12,786	75	926	1249	1811	2582	3547	4905	5737	12,861	1.38	3.12
Having no condition	27,113	3568	1906	25,949	0	1215	1585	2265	3223	4438	5953	7083	25,949	1.64	5.90
Having ≥ 1 condition	15,356	3219 *	1722	20,999	5	1116	1436	2042	2890	4027	5356	6404	21,004	1.61	5.54

^a^ Summary statistic: N = sample size; SD = standard deviation. ^b^ Percentiles and extremes: Min = minimum; percentiles (P5, P10, P25, P50, P75, P90, and P95); Max = maximum. ^c^ Shape parameters: Skew = skewness; Kurt = kurtosis. ^d^ “With” and “Without” indicate participants’ health condition. * Mean sodium intake compared using Welch’s *t*-test for paired subgroups (adults vs. children, males vs. boys, females vs. girls, and with vs. without health conditions), indicating *p* < 0.0001.

**Table 3:** Results from regression Model 4 examining temporal and age-related trends in sodium intake for 16 select groups *.

Group	Temporal Trend Across Cycle ‡		Age-Related Effect	
	Pattern	Turning Point (in Cycle)	Pattern	Turning Point (in Years)
Children (5–17)	No		ID Increase	†
Adults (18 and older)	ID Slow incline	6.80	ID-∩	23.14
Boys	No		IA Fast increase	†
Male adults	ID Slow incline	5.42	ID-∩	26.60
Girls	DD Fast decline	†	ID Slow increase	12.65
Female adults	No		ID Decline	†
Hypertension (Without)	ID Slow incline	6.36	ID-∩ Rise-then-fall	23.53
Hypertension (With)	ID Slow incline	7.05	ID Decline	†
Heart disease (Without)	ID Slow incline	6.62	ID-∩ Rise-then-fall	24.05
Heart disease (With)	No		No	
Heart attack (Without)	ID Slow incline	6.36	ID-∩ Rise-then-fall	23.59
Heart attack (With)	No		No	
Stroke (Without)	ID Slow incline	6.78	ID-∩ Rise-then-fall	23.66
Stroke (With)	No		No	
Having no condition	ID Slow incline	5.99	ID-∩ Rise-then-fall	24.27
Having ≥1 condition	ID Slow incline	7.81	ID Slow decrease	†

* Interpretations of patterns and turning points are presented in Table 1. † Outside of the meaningful range. ‡ NHANES cycles are coded from 0 to 7, corresponding to survey years 2003–2004 (cycle 0), 2005–2006 (cycle 1), 2007–2008 (cycle 2), 2009–2010 (cycle 3), 2011–2012 (cycle 4), 2013–2014 (cycle 5), 2015–2016 (cycle 6), and 2017–2018 (cycle 7).

## Data Availability

The data presented in this study are openly available in the National Health and Nutrition Examination Survey (NHANES) database.

## Supplementary Materials

The following supporting information can be downloaded at: https://www.mdpi.com/article/10.3390/nu17121975/s1, Table S1: Descriptive summary of sodium intake among children (Ages 5–17) and adults (Ages 18 and older) stratified by sex and in adults based on health conditions, by NHANES Cycle (2003–2018), based on unweighted data; Table S2: Results from Regression Models Examining Temporal Trends in Sodium Intake (Models 1–3), Using Weighted and Unweighted Individual-Level Data for Specific Population Groups; Table S3: Results from Regression Model 4 Examining Temporal and Age-Related Trends in Sodium Intake, Using Weighted and Unweighted Individual-Level Data for Specific Population Groups; Table S4: Temporal Trends in Sodium Intake, Based on Unweighted Population-Level Data Among Children (Ages 5–17 Years) and Adults (Ages 18 and Older), Adjusted for Survey Cycle; Table S5: Sensitivity analysis: Descriptive summary of sodium intake among children (Ages 5–17) and adults (Ages 18 and older) stratified by sex and in adults based on health conditions, pooled across eight NHANES cycles (2003–2018), based on weighted data; Table S6: Sensitivity analysis: Descriptive summary of sodium intake among children (Ages 5–17) and adults (Ages 18 and older) stratified by sex and in adults based on health conditions, by NHANES Cycle (2003–2018), based on weighted data; Table S7: Sensitivity analysis: Temporal Trends in Sodium Intake, Based on Weighted Population-Level Data Among Children (Ages 5–17 Years) and Adults (Ages 18 and Older), Adjusted for Survey Cycle.
