# Supplementary material for: Beyond the Average: Trends in Extreme Sodium Intake in the U.S. Population, 2003–2018
Source: Nutrients. 2025 Jun 11;17(12):1975. doi: 10.3390/nu17121975 (PMC12196499; doi:10.3390/nu17121975)
Supplement: Supplementary file 1 [file nutrients-17-01975-s001.zip › nutrients-3672551-supplementary.pdf]

Beyond the Average: Trends in Extreme Sodium Intake in the U.S. Population, 2003–2018

Yutong Chen[1], Jingyan Wang[1], Kristin E. Leonberg[1], Kenneth Kwan Ho Chui[2], Lynne Ausman[1], Elena N. Naumova[1,] *
[1] Division of Nutrition Epidemiology and Data Science, Friedman School of Nutrition Science and Policy, Tufts University, Boston, MA, US
[2] Department of Public Health and Community Medicine, Tufts University School of Medicine, Boston, MA, USA
* Correspondence: Elena.Naumova@tufts.edu

Supplementary Materials

Table S1. Descriptive summary of sodium intake among children (Ages 5–17) and adults (Ages 18 and older) stratified by sex and in adults based on health conditions, by NHANES Cycle (2003–2018), based on unweighted data

Group [a]	Yr[b]	N [c]	Mean	SD[d]	Range	Min[e]	P05[f]	P10 [f]	P25 [f]	P50 [f]	P75 [f]	P90 [f]	P95 [f]	Max[g]	Skew[h]	Kurt[i]
Children (5-17)																
	2003	2,698	3,266	1,660	12,131	0	1,205	1,521	2,120	2,937	4,105	5,418	6,302	12,131	1.23	2.32
	2005	2,778	3,260	1,750	15,503	195	1,172	1,479	2,105	2,963	3,980	5,337	6,314	15,698	1.91	6.89
	2007	2,175	3,051	1,571	20,218	107	1,142	1,461	2,008	2,764	3,788	4,964	5,958	20,325	1.87	9.18
	2009	2,306	3,169	1,544	15,713	263	1,282	1,604	2,141	2,881	3,840	5,047	6,012	15,976	1.74	6.16
	2011	2,187	3,147	1,493	14,150	115	1,205	1,532	2,157	2,900	3,855	5,039	6,047	14,265	1.33	3.40
	2013	2,186	3,093	1,555	17,475	17	1,189	1,482	2,026	2,813	3,807	5,009	6,005	17,492	1.64	5.93
	2015	2,135	3,151	1,602	16,414	0	1,186	1,465	2,096	2,869	3,845	5,065	6,218	16,414	1.66	5.55
	2017	1,729	3,045	1,531	11,554	42	1,175	1,450	1,980	2,725	3,815	5,058	5,970	11,596	1.34	2.99
Adults (18 and older)																
	2003	4,986	3,355	1,827	15,047	15	1,066	1,408	2,071	3,039	4,203	5,749	6,775	15,062	1.30	2.80
	2005	5,060	3,414	1,831	18,022	31	1,144	1,507	2,151	3,087	4,312	5,659	6,738	18,053	1.52	4.65
	2007	5,690	3,295	1,870	21,248	0	1,056	1,406	2,039	2,934	4,114	5,577	6,671	21,248	1.85	7.64
	2009	6,052	3,462	1,787	17,613	25	1,269	1,598	2,243	3,114	4,286	5,692	6,784	17,638	1.53	4.31
	2011	5,076	3,565	1,849	20,176	7	1,343	1,648	2,295	3,210	4,409	5,876	6,992	20,183	1.62	5.33
	2013	5,356	3,507	1,863	21,370	29	1,238	1,589	2,263	3,168	4,362	5,789	6,957	21,399	1.80	7.25
	2015	5,266	3,487	1,818	16,536	34	1,198	1,567	2,235	3,175	4,339	5,805	6,917	16,570	1.42	3.73
	2017	4,983	3,456	1,940	25,949	0	1,174	1,485	2,137	3,102	4,311	5,886	6,936	25,949	1.99	9.81
Boys																
	2003	1,354	3,531	1,772	12,131	0	1,291	1,614	2,338	3,196	4,467	5,823	6,793	12,131	1.15	2.05
	2005	1,379	3,617	1,966	15,503	195	1,351	1,690	2,359	3,263	4,341	5,918	7,208	15,698	1.91	6.21
	2007	1,113	3,293	1,714	20,218	107	1,289	1,542	2,168	2,926	4,060	5,450	6,494	20,325	1.97	10.18
	2009	1,186	3,445	1,694	14,523	442	1,371	1,719	2,314	3,099	4,179	5,597	6,839	14,965	1.49	3.73
	2011	1,117	3,380	1,596	14,018	247	1,344	1,665	2,298	3,091	4,144	5,480	6,430	14,265	1.31	3.39
	2013	1,119	3,397	1,726	17,318	174	1,336	1,671	2,198	3,048	4,165	5,551	6,580	17,492	1.68	5.92
	2015	1,077	3,404	1,745	16,146	268	1,252	1,560	2,257	3,059	4,163	5,701	6,702	16,414	1.59	4.92
	2017	853	3,356	1,664	11,447	149	1,320	1,624	2,159	3,066	4,161	5,579	6,430	11,596	1.26	2.62
Male																
	2003	2,395	3,846	2,031	15,027	35	1,220	1,635	2,402	3,490	4,866	6,498	7,557	15,062	1.14	2.03
	2005	2,425	3,935	2,066	17,924	129	1,347	1,755	2,504	3,595	4,950	6,530	7,821	18,053	1.37	3.36
	2007	2,809	3,808	2,054	21,248	0	1,277	1,681	2,408	3,424	4,745	6,373	7,593	21,248	1.66	6.17
	2009	2,946	4,033	2,013	17,454	184	1,466	1,896	2,640	3,715	5,025	6,508	7,834	17,638	1.34	3.18
	2011	2,535	4,093	1,986	15,178	7	1,556	2,014	2,723	3,760	5,057	6,629	7,647	15,185	1.32	2.95
	2013	2,558	4,041	2,066	21,370	29	1,524	1,919	2,604	3,681	4,965	6,690	7,830	21,399	1.61	5.45
	2015	2,543	3,974	1,983	15,908	82	1,413	1,836	2,598	3,652	4,915	6,521	7,723	15,990	1.25	2.76
	2017	2,426	3,962	2,161	25,927	22	1,353	1,753	2,526	3,614	4,941	6,610	7,802	25,949	1.99	9.95
Girls																
	2003	1,344	2,999	1,494	10,141	192	1,109	1,435	1,965	2,741	3,701	4,909	5,891	10,333	1.24	2.31

	2005	1,399	2,909	1,422	11,436	269	1,087	1,363	1,912	2,698	3,622	4,658	5,442	11,705	1.39	4.11
	2007	1,062	2,796	1,360	10,783	149	1,062	1,377	1,885	2,566	3,437	4,434	5,171	10,932	1.44	4.11
	2009	1,120	2,877	1,307	15,713	263	1,233	1,539	2,048	2,679	3,438	4,423	5,151	15,976	2.04	11.81
	2011	1,070	2,904	1,336	9,053	115	1,110	1,418	2,027	2,687	3,554	4,522	5,397	9,168	1.21	2.63
	2013	1,067	2,775	1,279	8,777	17	1,097	1,347	1,861	2,607	3,363	4,442	5,205	8,794	1.09	1.94
	2015	1,058	2,893	1,397	13,096	0	1,140	1,389	1,975	2,672	3,514	4,580	5,372	13,096	1.60	5.61
	2017	876	2,742	1,322	9,778	42	1,148	1,341	1,838	2,468	3,451	4,503	5,073	9,820	1.25	2.56
Female																
	2003	2,591	2,900	1,476	11,731	15	974	1,278	1,857	2,660	3,689	4,717	5,642	11,746	1.17	2.51
	2005	2,635	2,934	1,426	17,394	31	1,012	1,325	1,927	2,708	3,701	4,820	5,473	17,425	1.21	4.93
	2007	2,881	2,794	1,513	18,561	5	893	1,225	1,793	2,549	3,473	4,587	5,414	18,566	2.02	10.97
	2009	3,106	2,920	1,334	13,675	25	1,168	1,444	2,010	2,709	3,642	4,626	5,332	13,700	1.23	3.61
	2011	2,541	3,037	1,528	20,050	133	1,156	1,483	2,010	2,800	3,721	4,818	5,746	20,183	2.14	12.72
	2013	2,798	3,018	1,498	20,959	45	1,104	1,382	2,007	2,784	3,755	4,825	5,633	21,004	1.86	10.92
	2015	2,723	3,033	1,516	16,536	34	1,074	1,403	2,004	2,766	3,782	4,859	5,824	16,570	1.48	5.11
	2017	2,557	2,976	1,560	14,473	0	1,076	1,354	1,909	2,694	3,718	4,891	5,843	14,473	1.59	5.22
Hypertension (Without)																
	2003	3,404	3,490	1,878	14,049	15	1,073	1,463	2,176	3,181	4,351	5,968	7,025	14,064	1.23	2.44
	2005	3,599	3,511	1,876	18,022	31	1,167	1,534	2,206	3,183	4,429	5,792	6,899	18,053	1.50	4.40
	2007	3,734	3,436	1,941	21,248	0	1,103	1,470	2,137	3,069	4,302	5,898	6,881	21,248	1.90	8.21
	2009	3,998	3,563	1,812	14,974	25	1,304	1,661	2,310	3,220	4,428	5,809	6,954	14,999	1.44	3.70
	2011	3,328	3,674	1,894	16,964	106	1,355	1,717	2,371	3,312	4,561	6,063	7,195	17,070	1.52	4.28
	2013	3,471	3,613	1,889	21,354	45	1,335	1,691	2,332	3,282	4,465	5,932	7,090	21,399	1.75	6.68
	2015	3,407	3,569	1,873	16,536	34	1,209	1,591	2,293	3,236	4,435	5,919	7,063	16,570	1.47	4.11
	2017	3,154	3,555	2,035	25,949	0	1,193	1,511	2,202	3,189	4,372	6,068	7,212	25,949	2.18	11.39
Hypertension (With)																
	2003	1,582	3,062	1,675	14,934	128	1,012	1,328	1,875	2,752	3,845	5,188	6,274	15,062	1.45	3.88
	2005	1,461	3,175	1,693	17,340	85	1,056	1,395	2,023	2,851	4,033	5,199	6,245	17,425	1.53	5.31
	2007	1,956	3,026	1,695	15,189	5	986	1,312	1,872	2,717	3,815	5,085	6,127	15,194	1.64	5.01
	2009	2,054	3,264	1,723	17,431	207	1,195	1,507	2,106	2,948	3,987	5,422	6,362	17,638	1.73	5.90
	2011	1,748	3,355	1,741	20,176	7	1,331	1,567	2,200	3,013	4,191	5,319	6,549	20,183	1.85	8.14
	2013	1,885	3,311	1,797	20,975	29	1,156	1,418	2,137	2,972	4,172	5,463	6,532	21,004	1.91	8.69
	2015	1,859	3,339	1,704	11,836	82	1,165	1,546	2,161	3,031	4,170	5,575	6,523	11,918	1.26	2.38
	2017	1,829	3,286	1,753	14,206	9	1,141	1,459	2,047	2,946	4,186	5,524	6,658	14,215	1.37	3.46
Heart disease (Without)																
	2003	4,753	3,378	1,838	15,047	15	1,067	1,414	2,091	3,062	4,235	5,795	6,796	15,062	1.30	2.79
	2005	4,883	3,434	1,844	18,022	31	1,150	1,512	2,158	3,098	4,335	5,686	6,766	18,053	1.52	4.61
	2007	5,467	3,306	1,882	21,248	0	1,051	1,405	2,046	2,938	4,134	5,608	6,694	21,248	1.86	7.65
	2009	5,817	3,478	1,798	17,613	25	1,271	1,600	2,251	3,127	4,303	5,722	6,809	17,638	1.52	4.30
	2011	4,904	3,578	1,858	20,176	7	1,341	1,654	2,299	3,229	4,425	5,894	7,038	20,183	1.62	5.31
	2013	5,153	3,523	1,871	21,354	45	1,257	1,602	2,275	3,186	4,372	5,826	6,969	21,399	1.81	7.33
	2015	5,053	3,499	1,832	16,536	34	1,191	1,564	2,242	3,180	4,356	5,826	6,954	16,570	1.42	3.72
	2017	4,762	3,468	1,954	25,949	0	1,182	1,494	2,141	3,111	4,316	5,920	6,981	25,949	2.02	9.93
Heart disease (With)																
	2003	233	2,876	1,490	7,860	484	1,001	1,355	1,786	2,475	3,595	4,923	5,824	8,344	1.10	1.21
	2005	177	2,869	1,333	7,410	349	893	1,234	2,031	2,699	3,520	4,682	5,357	7,759	0.69	0.61
	2007	223	3,022	1,548	11,474	5	1,186	1,416	1,848	2,801	3,821	4,794	6,033	11,479	1.40	3.92
	2009	235	3,048	1,433	8,117	515	1,198	1,579	2,013	2,848	3,819	4,790	5,725	8,632	1.15	1.74
	2011	172	3,167	1,526	10,943	251	1,388	1,614	2,244	2,867	3,777	5,141	5,732	11,194	1.63	4.78
	2013	203	3,086	1,596	9,110	29	993	1,332	1,980	2,848	4,070	5,056	5,560	9,139	1.03	1.46
	2015	213	3,215	1,447	8,009	763	1,434	1,653	2,157	3,020	4,006	5,036	6,046	8,772	0.93	0.92
	2017	221	3,202	1,604	8,404	75	1,091	1,340	2,047	2,905	4,156	5,345	6,270	8,479	0.72	0.36
Heart attack (Without)																
	2003	4,743	3,376	1,836	15,047	15	1,066	1,416	2,092	3,060	4,230	5,802	6,804	15,062	1.29	2.74
	2005	4,870	3,435	1,841	18,022	31	1,149	1,516	2,163	3,099	4,335	5,678	6,765	18,053	1.52	4.65
	2007	5,435	3,324	1,878	21,248	0	1,082	1,435	2,059	2,961	4,152	5,615	6,706	21,248	1.87	7.72
	2009	5,810	3,473	1,785	17,613	25	1,282	1,604	2,249	3,125	4,300	5,714	6,790	17,638	1.50	4.16
	2011	4,900	3,581	1,855	20,176	7	1,344	1,663	2,304	3,230	4,429	5,895	7,036	20,183	1.61	5.24
	2013	5,154	3,520	1,862	21,354	45	1,249	1,599	2,273	3,184	4,377	5,807	6,960	21,399	1.78	7.15
	2015	5,048	3,500	1,823	16,536	34	1,209	1,572	2,245	3,186	4,357	5,809	6,945	16,570	1.43	3.78
	2017	4,756	3,467	1,952	25,949	0	1,183	1,498	2,142	3,112	4,309	5,906	6,979	25,949	2.03	10.00
Heart attack (With)																
	2003	243	2,926	1,577	11,533	483	1,100	1,327	1,756	2,639	3,698	4,949	5,816	12,016	1.51	4.41

	2005	190	2,878	1,478	8,869	509	1,086	1,322	1,807	2,596	3,516	4,773	5,493	9,378	1.19	2.12
	2007	255	2,683	1,593	11,474	5	660	987	1,557	2,414	3,460	4,557	5,663	11,479	1.40	3.83
	2009	242	3,195	1,823	13,314	650	971	1,335	1,999	2,903	3,994	5,114	5,985	13,964	2.12	8.07
	2011	176	3,097	1,608	13,298	251	1,230	1,511	2,151	2,807	3,760	4,977	5,706	13,549	2.13	9.62
	2013	202	3,159	1,860	15,696	29	1,071	1,438	2,016	2,820	3,830	5,010	6,494	15,725	2.37	10.79
	2015	218	3,207	1,682	10,220	505	1,081	1,465	2,111	2,741	3,942	5,678	6,299	10,725	1.15	1.70
	2017	227	3,219	1,668	8,171	175	1,037	1,300	1,980	2,881	4,349	5,528	6,396	8,346	0.71	0.03
Stroke (Without)																
	2003	4,806	3,378	1,837	15,047	15	1,072	1,421	2,091	3,069	4,228	5,811	6,810	15,062	1.30	2.77
	2005	4,891	3,441	1,838	18,022	31	1,160	1,521	2,170	3,108	4,340	5,678	6,762	18,053	1.52	4.66
	2007	5,458	3,320	1,881	21,248	0	1,073	1,419	2,051	2,969	4,143	5,617	6,705	21,248	1.86	7.67
	2009	5,849	3,478	1,792	17,613	25	1,271	1,600	2,250	3,134	4,301	5,718	6,809	17,638	1.51	4.24
	2011	4,887	3,589	1,860	20,176	7	1,345	1,663	2,313	3,238	4,437	5,899	7,041	20,183	1.62	5.31
	2013	5,186	3,526	1,863	21,370	29	1,259	1,609	2,280	3,191	4,384	5,810	6,958	21,399	1.81	7.37
	2015	5,082	3,503	1,825	16,536	34	1,217	1,585	2,246	3,185	4,360	5,827	6,944	16,570	1.43	3.76
	2017	4,754	3,478	1,948	25,949	0	1,186	1,510	2,147	3,135	4,323	5,919	6,970	25,949	2.02	10.04
Stroke (With)																
	2003	180	2,715	1,395	8,599	332	949	1,138	1,707	2,446	3,357	4,623	5,030	8,931	1.09	1.83
	2005	169	2,640	1,413	7,719	424	814	1,015	1,689	2,341	3,329	4,519	5,193	8,143	1.12	1.69
	2007	232	2,713	1,486	9,594	115	714	1,145	1,754	2,555	3,260	4,643	5,425	9,709	1.26	2.63
	2009	203	2,980	1,565	12,285	576	1,116	1,518	1,952	2,687	3,539	4,941	5,755	12,861	2.01	7.75
	2011	189	2,938	1,386	8,170	707	1,239	1,409	2,019	2,630	3,544	4,967	5,410	8,877	1.21	1.98
	2013	170	2,914	1,753	10,498	381	908	1,177	1,752	2,607	3,525	4,923	6,463	10,879	1.69	3.85
	2015	184	3,067	1,592	8,614	82	1,014	1,258	1,865	2,717	3,933	5,301	5,951	8,696	0.94	1.07
	2017	229	2,996	1,720	9,176	75	1,027	1,228	1,722	2,644	3,696	5,379	6,655	9,251	1.21	1.27
Having no condition																
	2003	3,257	3,514	1,883	14,049	15	1,079	1,473	2,198	3,201	4,409	6,019	7,041	14,064	1.21	2.35
	2005	3,473	3,531	1,886	18,022	31	1,177	1,546	2,219	3,195	4,440	5,823	6,939	18,053	1.49	4.39
	2007	3,585	3,458	1,950	21,248	0	1,111	1,478	2,152	3,091	4,344	5,916	6,910	21,248	1.90	8.24
	2009	3,875	3,582	1,818	14,974	25	1,331	1,689	2,326	3,240	4,452	5,828	6,968	14,999	1.44	3.69
	2011	3,239	3,686	1,899	16,964	106	1,358	1,722	2,380	3,331	4,568	6,078	7,218	17,070	1.52	4.26
	2013	3,364	3,625	1,882	21,354	45	1,336	1,697	2,340	3,294	4,480	5,963	7,090	21,399	1.72	6.49
	2015	3,284	3,588	1,886	16,536	34	1,213	1,614	2,301	3,242	4,459	5,948	7,120	16,570	1.47	4.08
	2017	3,036	3,566	2,049	25,949	0	1,191	1,519	2,209	3,205	4,372	6,094	7,226	25,949	2.20	11.43
Having >=1 condition																
	2003	1,729	3,055	1,674	14,934	128	1,006	1,327	1,875	2,750	3,830	5,170	6,249	15,062	1.48	4.04
	2005	1,587	3,158	1,677	17,340	85	1,058	1,395	2,024	2,824	3,994	5,199	6,194	17,425	1.52	5.23
	2007	2,105	3,016	1,691	15,189	5	985	1,306	1,870	2,722	3,799	5,031	6,125	15,194	1.65	5.07
	2009	2,177	3,247	1,710	17,431	207	1,183	1,491	2,106	2,930	3,974	5,386	6,350	17,638	1.72	5.86
	2011	1,837	3,350	1,737	20,176	7	1,332	1,573	2,201	3,001	4,165	5,320	6,544	20,183	1.85	8.03
	2013	1,992	3,306	1,812	20,975	29	1,143	1,420	2,133	2,964	4,169	5,451	6,546	21,004	1.97	9.01
	2015	1,982	3,321	1,689	11,836	82	1,158	1,519	2,158	3,034	4,142	5,539	6,453	11,918	1.25	2.40
	2017	1,947	3,285	1,743	14,206	9	1,149	1,456	2,050	2,942	4,189	5,512	6,648	14,215	1.35	3.35

a. "With" and "Without" indicates participants' health conditions.
b. Yr represents Cycle.
c. N = sample size.
d. SD = standard deviation.
e. Min = minimum.
f. P represents percentiles (P5, P10, P25, P50, P75, P90, and P95).
g. Max = maximum.
h. Skew = skewness.
i. Kurt = kurtosis.

Table S2. Results from Regression Models Examining Temporal Trends in Sodium Intake (Models 1–3), Using Weighted and Unweighted Individual-Level Data for Specific Population Groups

**Group [a]**			**Weighted**			**Unweighted**		
	**N**	**Term**	**Model 1**	**Model 2**	**Model 3**	**Model 1**	**Model 2**	**Model 3**
Children (5-17)	18,194	Intercept	3294.06 (38.96)***	3300.57 (43.65)***	2405.89 (58.09)***	3237.42 (20.54)***	3261.45 (26.15)***	2398.1 (45.65)***
		Cycle	-32.85 (9.16)***	-39.37 (34.7)*	-41.4 (33.42)*	-24.82 (5.2)***	-51.22 (18.53)***	-7.57 (18.37)
		Cycle²		0.93 (5.13)	1.09 (4.95)		3.92 (2.64)*	-1.19 (2.61)
		Age			81.02 (5.05)***			72.2 (3.15)***
Adults (18 and older)	42,469	Intercept	3510.74 (27.77)***	3475.16 (32.29)***	4253.98 (45.88)***	3364.23 (16.57)***	3324.28 (21.93)***	4172.45 (30.57)***
		Cycle	6.76 (6.27)*	41.27 (18.97)***	43.03 (18)***	22.17 (3.99)***	60.72 (14.44)***	63.42 (14.18)***
		Cycle²		-4.87 (2.7)**	-4.41 (2.51)**		-5.49 (1.98)***	-4.99 (1.94)***
		Age			-17.15 (0.64)***			-18.2 (0.47)***
Boys	9,198	Intercept	3608.69 (53.95)***	3583.29 (69.16)***	2079.13 (76.86)***	3524.01 (31.69)***	3562.62 (40.37)***	2146.47 (69.35)***
		Cycle	-34.86 (12.87)***	-9.05 (49.41)	2.11 (45.16)	-26.61 (8.04)***	-68.84 (28.52)***	12.06 (27.81)
		Cycle²		-3.69 (6.87)	-5.15 (6.32)*		6.28 (4.07)*	-3.29 (3.96)*
		Age			135.09 (6.8)***			117.12 (4.74)***
Male	20,637	Intercept	4155.62 (39.35)***	4104.04 (46.76)***	5002.45 (72.28)***	3887.17 (26.37)***	3816.96 (35)***	4926.67 (48.62)***
		Cycle	-3.18 (9.25)	46.82 (29.32)*	47.82 (29.24)**	21.43 (6.36)***	88.69 (22.95)***	83.82 (22.4)***
		Cycle²		-7.06 (4.17)**	-6.34 (4.07)*		-9.57 (3.14)***	-7.9 (3.06)***
		Age			-20.12 (1.07)***			-23.44 (0.73)***
Girls	8,996	Intercept	2971.18 (47.74)***	3000.12 (54.34)***	2685.34 (83.18)***	2945.11 (24.99)***	2963.56 (31.8)***	2676.17 (56.1)***
		Cycle	-30.89 (11.44)***	-59.41 (40.11)*	-63.31 (39.81)*	-23.2 (6.32)***	-43.58 (22.62)**	-31 (22.66)*
		Cycle²		4.05 (6.07)	4.48 (6)*		3.02 (3.22)*	1.57 (3.22)
		Age			28.76 (5.96)***			24.31 (3.91)***
Female	21,832	Intercept	2914.5 (26.74)***	2898.73 (34)***	3441.15 (50.45)***	2871.76 (18.47)***	2869.4 (24.4)***	3506.33 (33.95)***
		Cycle	14.54 (6.45)***	29.86 (20.98)*	31.74 (19.69)*	22.34 (4.45)***	24.63 (16.12)*	31.24 (15.86)***
		Cycle²		-2.16 (2.97)*	-1.95 (2.81)*		-0.33 (2.21)	-0.51 (2.17)
		Age			-11.77 (0.7)***			-13.88 (0.52)***
		Cycle	14.54 (6.45)***	29.86 (20.98)*	31.74 (19.69)*	22.34 (4.45)***	24.63 (16.12)*	31.24 (15.86)***
		Cycle²		-2.16 (2.97)*	-1.95 (2.81)*		-0.33 (2.21)	-0.51 (2.17)

		Age			-11.77 (0.7)***			-13.88 (0.52)***
Hypertension (Without)	28,095	Intercept	3610.85 (31.61)***	3581.37 (39.84)***	4195.37 (56.09)***	3491.38 (20.64)***	3453.92 (27.22)***	4093.27 (37.31)***
		Cycle	-1.62 (6.95)	27.04 (23.83)*	25.54 (23.41)*	17.05 (5.04)***	53.89 (18.16)***	55.54 (17.97)***
		Cycle²		-4.05 (3.24)*	-3.4 (3.15)*		-5.28 (2.5)***	-4.78 (2.48)**
		Age			-15.01 (0.83)***			-15.77 (0.64)***
Hypertension (With)	14,374	Intercept	3272.38 (46.45)***	3223.59 (55.49)***	4623.09 (97.38)***	3084.81 (27.33)***	3031.71 (36.5)***	4510.95 (65.44)***
		Cycle	28.74 (10.88)***	75.83 (33.59)***	87.45 (32.54)***	40.21 (6.43)***	89.56 (23.39)***	78.08 (22.83)***
		Cycle²		-6.63 (4.61)*	-7.34 (4.54)*		-6.93 (3.16)***	-5.59 (3.08)**
		Age			-24.91 (1.3)***			-24.54 (0.91)***
Heart disease (Without)	40,792	Intercept	3525.28 (27.91)***	3490.41 (32.32)***	4255.97 (46.15)***	3384.25 (17.03)***	3345.86 (22.57)***	4174.89 (31.28)***
		Cycle	5.91 (6.31)*	39.63 (18.92)***	43.32 (17.86)***	20.93 (4.11)***	57.89 (14.84)***	63.2 (14.59)***
		Cycle²		-4.76 (2.7)**	-4.58 (2.51)**		-5.27 (2.03)***	-5.09 (2)***
		Age			-17.18 (0.65)***			-18.21 (0.48)***
Heart disease (With)	1,677	Intercept	3097.63 (94.98)***	3098.66 (116.84)***	4520.63 (395.58)***	2883.51 (66.09)***	2855.18 (85.25)***	4110.03 (244.95)***
		Cycle	30.57 (25.02)*	29.47 (86.88)	39.12 (85.12)	50.56 (15.76)***	79.46 (57.14)*	67.88 (56.69)*
		Cycle²		0.16 (12.98)	-0.29 (12.97)		-4.13 (7.84)	-2.64 (7.78)
		Age			-21.57 (5.64)***			-18.1 (3.32)***
Heart attack (Without)	40,716	Intercept	3528.47 (27.75)***	3489.02 (31.99)***	4252.82 (46.66)***	3388.14 (16.99)***	3348.01 (22.52)***	4166.94 (31.17)***
		Cycle	4.5 (6.37)*	42.62 (19.01)***	46.21 (18.17)***	20.27 (4.1)***	58.91 (14.81)***	64.75 (14.56)***
		Cycle²		-5.38 (2.72)***	-5.1 (2.56)***		-5.5 (2.03)***	-5.37 (1.99)***
		Age			-17.13 (0.67)***			-18.01 (0.48)***
Heart attack (With)	1,753	Intercept	3044.76 (116.3)***	3151.85 (144.91)***	4673.66 (398.91)***	2832.88 (71.36)***	2842.6 (92.69)***	4398.35 (244.28)***
		Cycle	60.51 (34.98)**	-56.69 (103.5)	-55.56 (97.57)	59.95 (17.19)***	50.08 (62.48)*	20.35 (61.83)
		Cycle²		16.98 (15.41)*	17.02 (14.93)*		1.41 (8.57)	4.75 (8.47)
		Age			-23.49 (5.36)***			-22.56 (3.28)***
Stroke (Without)	40,913	Intercept	3534.51 (27.27)***	3497.3 (32.07)***	4240.74 (46.96)***	3389.15 (16.94)***	3350.61 (22.42)***	4159.98 (31.16)***
		Cycle	5.26 (6.24)*	41.37 (18.91)***	43.61 (18.16)***	21.24 (4.09)***	58.48 (14.77)***	62.39 (14.53)***
		Cycle²		-5.1 (2.68)**	-4.73 (2.52)**		-5.31 (2.02)***	-4.96 (1.99)***

		Age			-16.58 (0.67)***			-17.69 (0.48)***
Stroke (With)	1,556	Intercept	2667.42 (102.49)***	2727.22 (139.66)***	3811.57 (254.38)***	2678.27 (72.45)***	2639.6 (97.05)***	3982.73 (224.26)***
		Cycle	65.18 (28.35)***	7.86 (95.9)	10.11 (92.09)	54.51 (17.07)***	90.89 (63.09)*	76.04 (62.27)*
		Cycle²		7.95 (14.08)	7.79 (13.57)		-5.06 (8.45)	-4.21 (8.34)
		Age			-16.95 (2.88)***			-19.73 (2.98)***
Having no condition	27,113	Intercept	3624.8 (30.74)***	3595.02 (37.81)***	4186.45 (57.09)***	3514.33 (21.13)***	3477.18 (27.89)***	4081.84 (38.17)***
		Cycle	-1.9 (6.84)	26.96 (22.85)*	27.93 (22.75)*	15.54 (5.16)***	51.96 (18.58)***	57.58 (18.4)***
		Cycle²		-4.08 (3.15)*	-3.75 (3.1)*		-5.22 (2.56)***	-5.17 (2.53)***
		Age			-14.77 (0.89)***			-15.39 (0.67)***
Having >=1 condition	15,356	Intercept	3265.87 (44.37)***	3222.05 (52.54)***	4599.79 (95.28)***	3071.43 (26.18)***	3022.68 (34.86)***	4491.33 (63.44)***
		Cycle	27.25 (10.33)***	69.84 (31.38)***	76.01 (30.07)***	41.07 (6.18)***	86.82 (22.48)***	70.28 (21.95)***
		Cycle²		-6 (4.3)*	-6.06 (4.21)*		-6.44 (3.04)***	-4.57 (2.97)*
		Age			-24.27 (1.3)***			-24.09 (0.88)***

* p-value <0.5; **p-value<0.1; ***p-value<0.05.

a. "With" and "Without" indicates participants' health conditions.

Table S3. Results from Regression Model 4 Examining Temporal and Age-Related Trends in Sodium Intake, Using Weighted and Unweighted Individual-Level Data for Specific Population Groups

Group [a]	N	Term	Weighted	Unweighted
Children (5-17)	18,194	Intercept	2064.4 (155.3)***	2070.07 (111.36)***
		Cycle	-41.64 (33.74)*	-8.52 (18.37)
		Cycle²	1.1 (5)	-1.1 (2.61)
		Age	150.96 (32.09)***	139.72 (21.15)***
		Age²	-3.17 (1.52)***	-3.06 (0.95)***
Adults (18 and older)	42,469	Intercept	3469.38 (97.15)***	3478.81 (60.81)***
		Cycle	41.54 (17.8)***	50.85 (14.19)***
		Cycle²	-4.1 (2.49)**	-3.74 (1.94)**
		Age	20.6 (4)***	16.54 (2.68)***
		Age²	-0.39 (0.04)***	-0.36 (0.03)***
Boys	9,198	Intercept	2288.53 (222.19)***	2210.81 (167.88)***
		Cycle	1.98 (45.03)	12.28 (27.81)
		Cycle²	-5.12 (6.3)*	-3.31 (3.96)*
		Age	92.2 (48.07)**	103.83 (31.92)***
		Age²	1.94 (2.28)*	0.6 (1.43)
Male	20,637	Intercept	3839.49 (157.79)***	3891.55 (95.86)***
		Cycle	46.44 (29.07)*	66.45 (22.36)***
		Cycle²	-5.9 (4.06)*	-6.13 (3.05)***
		Age	36.44 (6.36)***	28.34 (4.2)***
		Age²	-0.6 (0.06)***	-0.53 (0.04)***

Girls	8,996	Intercept	1828.87 (202.82)***	1868.29 (137.67)***
		Cycle	-65.08 (39.7)**	-33.02 (22.61)*
		Cycle^2	4.72 (6.01)*	1.8 (3.21)
		Age	204.18 (38.48)***	190.1 (26.11)***
		Age^2	-7.93 (1.81)***	-7.52 (1.17)***
Female	21,832	Intercept	3011.36 (101.75)***	3092.57 (68.07)***
		Cycle	30.63 (19.6)*	23.22 (15.89)*
		Cycle^2	-1.77 (2.8)	0.28 (2.18)
		Age	8.71 (4.15)***	6.87 (3.01)***
		Age^2	-0.21 (0.04)***	-0.21 (0.03)***
Hypertension (Without)	28,095	Intercept	3569.64 (117.73)***	3489.56 (73.06)***
		Cycle	23.05 (23.26)*	41.49 (18)***
		Cycle^2	-2.95 (3.14)*	-3.26 (2.48)*
		Age	16.89 (5.19)***	16.4 (3.41)***
		Age^2	-0.35 (0.05)***	-0.35 (0.04)***
Hypertension (With)[a]	14,374	Intercept	3564.86 (234.14)***	3748.52 (156.72)***
		Cycle	86.07 (32.67)***	73.19 (22.82)***
		Cycle^2	-7.13 (4.55)*	-5.19 (3.08)**
		Age	17.5 (8.54)***	6.2 (5.82)*
		Age^2	-0.39 (0.07)***	-0.28 (0.05)***
Heart disease (Without)[a]	40,792	Intercept	3449.24 (98.96)***	3455.42 (62.1)***
		Cycle	41.75 (17.57)***	49.99 (14.59)***
		Cycle^2	-4.26 (2.48)**	-3.78 (2)**
		Age	21.93 (4.11)***	18.11 (2.75)***
		Age^2	-0.41 (0.04)***	-0.38 (0.03)***
Heart disease (With)[a]	1,677	Intercept	3191.1 (1425)***	3121.77 (831.98)***
		Cycle	44.44 (84.51)	68.93 (56.69)*
		Cycle^2	-1.02 (12.93)	-2.85 (7.78)
		Age	22.54 (44.61)	14.8 (26.68)
		Age^2	-0.35 (0.34)*	0 (0)***
Heart attack (Without)[a]	40,716	Intercept	3462.67 (97.34)***	3468.14 (61.97)***
		Cycle	44.76 (17.96)***	52.22 (14.56)***
		Cycle^2	-4.77 (2.53)**	-4.11 (1.99)***
		Age	21.11 (4.03)***	17.23 (2.74)***
		Age^2	-0.4 (0.04)***	-0.37 (0.03)***
Heart attack (With)[a]	1,753	Intercept	3061.85 (1393)***	3365.42 (802.28)***
		Cycle	-48.87 (98.39)	20.3 (61.81)
		Cycle^2	15.76 (15.05)*	4.52 (8.47)
		Age	32.39 (43.61)*	12.95 (26.48)
		Age^2	-0.46 (0.34)*	-0.29 (0.21)*
Stroke (Without)[a]	40,913	Intercept	3470.42 (98.83)***	3471.94 (61.97)***
		Cycle	42 (17.95)***	49.7 (14.53)***
		Cycle^2	-4.39 (2.5)**	-3.67 (1.99)**
		Age	20.72 (4.12)***	17 (2.74)***
		Age^2	-0.39 (0.04)***	-0.36 (0.03)***
Stroke (With)[a]	1,556	Intercept	2772.87 (794.16)***	3188.36 (652.01)***
		Cycle	15.33 (92.97)	74.96 (62.27)*
		Cycle^2	6.88 (13.62)	-4.28 (8.33)
		Age	21.14 (26.53)*	8.6 (22.04)
		Age^2	-0.33 (0.22)*	-0.24 (0.18)*

Having no condition	27,113	Intercept	3545.72 (119.12)***	3467.95 (75.06)***
		Cycle	25.97 (22.62)*	43.98 (18.43)***
		Cycle²	-3.34 (3.08)*	-3.67 (2.54)*
		Age	18.19 (5.28)***	17.59 (3.54)***
		Age²	-0.37 (0.05)***	-0.36 (0.04)***
Having >=1 condition	15,356	Intercept	3563.9 (226.27)***	3757.07 (152.26)***
		Cycle	74.86 (30.12)***	65.44 (21.95)***
		Cycle²	-5.91 (4.21)*	-4.19 (2.97)*
		Age	17.16 (8.16)***	5.45 (5.64)*
		Age²	-0.38 (0.07)***	-0.27 (0.05)***

* p-value <0.5; **p-value<0.1; ***p-value<0.05.

a. "With" and "Without" indicates participants' health conditions.

Table S4. Temporal Trends in Sodium Intake, Based on Unweighted Population-Level Data Among Children (Ages 5–17 Years) and Adults (Ages 18 and Older), Adjusted for Survey Cycle

			**P05[a]**	**P10[a]**	**P25[a]**	**P50[a]**	**P75[a]**	**P90[a]**	**P95[a]**
**Group[c]**	**Model**	**Term**	**β (SE)[b]**	**β (SE)[b]**	**β (SE)[b]**	**β (SE)[b]**	**β (SE)[b]**	**β (SE)[b]**	**β (SE)[b]**
Children (5-17)	Model 1	Intercept	1,197.67 (28.37)***	1,523.25 (33.66)***	2,118.92 (41.50)***	2,931.00 (45.02)***	3,989.08 (53.24)***	5,273.5 (86.36)***	6,212.75 (89.74)***
		Cycle	-0.90 (6.78)	-6.86 (8.05)*	-11.37 (9.92)*	-21.29 (10.76)**	-31.35 (12.73)***	-44.68 (20.64)**	-31.29 (21.45)*
	Model 2	Intercept	1,180.58 (38.73)***	1,491.25 (42.59)***	2,090.13 (55.77)***	2,923.92 (64.11)***	4,072.63 (48.92)***	5,414.54 (74.52)***	6,306.58 (110.24)***
		Cycle	16.18 (25.85)	25.14 (28.42)*	17.42 (37.22)	-14.20 (42.78)	-114.89 (32.65)***	-185.72 (49.73)***	-125.12 (73.57)*
		Cycle²	-2.44 (3.55)	-4.57 (3.90)*	-4.11 (5.11)*	-1.01 (5.88)	11.93 (4.48)***	20.15 (6.83)***	13.40 (10.11)*
Adults (18 and older)	Model 1	Intercept	1,117.42 (59.78)***	1,466.08 (55.14)***	2,112.33 (56.64)***	3,033.67 (50.76)***	4,218.75 (54.52)***	5,649.58 (54.57)***	6,717.58 (53.48)***
		Cycle	19.60 (14.29)*	17.12 (13.18)*	19.12 (13.54)*	19.99 (12.14)*	20.93 (13.03)*	29.87 (13.05)**	36.76 (12.79)***
	Model 2	Intercept	1,037.42 (64.67)***	1,387.33 (56.47)***	2,039.25 (62.86)***	3,005.46 (69.79)***	4,191.5 (75.52)***	5,690.04 (72.67)***	6,712.08 (76.29)***
		Cycle	99.60 (43.16)**	95.87 (37.69)**	92.20 (41.95)**	48.20 (46.58)*	48.18 (50.40)*	-10.59 (48.50)	42.26 (50.92)*
		Cycle²	-11.43 (5.93)*	-11.25 (5.18)**	-10.44 (5.76)*	-4.03 (6.40)	-3.89 (6.92)	5.78 (6.66)*	-0.79 (6.99)
Boys	Model 1	Intercept	1,326.67 (27.17)***	1,645.92 (43.10)***	2,331.75 (42.30)***	3,159.00 (62.71)***	4,324.67 (71.00)***	5,746.00 (101.78)***	6,902.08 (150.10)***
		Cycle	-2.12 (6.49)	-2.94 (10.30)	-20.11 (10.11)**	-18.71 (14.99)*	-32.76 (16.97)*	-31.04 (24.33)*	-62.17 (35.88)*
	Model 2	Intercept	1,302.92 (35.09)***	1,619.13 (58.66)***	2,329.13 (60.39)***	3,212.50 (81.44)***	4,433.42 (67.37)***	5,872.88 (115.39)***	6,939.75 (212.77)***
		Cycle	21.63 (23.42)*	23.85 (39.14)	-17.48 (40.30)	-72.21 (54.35)*	-141.51 (44.96)***	-157.91 (77.01)**	-99.83 (142.00)
		Cycle²	-3.39 (3.22)*	-3.83 (5.38)	-0.38 (5.54)	7.64 (7.47)*	15.54 (6.18)**	18.13 (10.58)*	5.38 (19.50)
Male	Model 1	Intercept	1,307.33 (69.97)***	1,725.17 (78.95)***	2,466.92 (66.66)***	3,534.33 (67.78)***	4,889.58 (63.96)***	6,469.42 (56.98)***	7,653.00 (70.5)***
		Cycle	24.90 (16.73)*	24.56 (18.87)*	23.92 (15.93)*	23.44 (16.20)*	12.40 (15.29)*	21.56 (13.62)*	20.82 (16.85)*
	Model 2	Intercept	1,193.08 (60.38)***	1,598.38 (70.06)***	2,373.04 (69.16)***	3,462.88 (83.02)***	4,846.83 (86.36)***	6,466.62 (81.36)***	7,627.29 (99.09)***
		Cycle	139.15	151.35	117.79	94.90	55.15	24.35	46.53

			(40.30)***	(46.76)***	(46.16)***	(55.41)*	(57.63)*	(54.29)	(66.13)
		Cycle^2	-16.32 (5.54)***	-18.11 (6.42)***	-13.41 (6.34)**	-10.21 (7.61)*	-6.11 (7.92)*	-0.40 (7.46)	-3.67 (9.08)
Girls	Model 1	Intercept	1,101.58 (34.82)***	1,431.92 (42.42)***	1,966.67 (51.96)***	2,719.33 (47.69)***	3,609.83 (60.33)***	4,688.42 (94.10)***	5,576.42 (138.63)***
		Cycle	6.19 (8.32)*	-8.80 (10.14)*	-7.94 (12.42)	-22.74 (11.40)**	-28.52 (14.42)**	-37.01 (22.50)*	-68.19 (33.14)**
	Model 2	Intercept	1,094.67 (49.50)***	1,399.71 (56.28)***	1,920.63 (66.92)***	2,697.83 (66.45)***	3,686.5 (67.60)***	4,845.46 (78.02)***	5,730.33 (166.43)***
		Cycle	13.11 (33.04)	23.41 (37.56)	38.10 (44.66)*	-1.24 (44.35)	-105.19 (45.11)**	-194.05 (52.07)***	-222.11 (111.07)**
		Cycle^2	-0.99 (4.54)	-4.60 (5.16)*	-6.58 (6.13)*	-3.07 (6.09)	10.95 (6.20)*	22.43 (7.15)***	21.99 (15.26)*
Female	Model 1	Intercept	988.58 (55.70)***	1,302.08 (51.71)***	1,881.67 (50.34)***	2,653.58 (49.26)***	3,621.25 (58.79)***	4,671.25 (62.01)***	5,437.00 (101.33)***
		Cycle	19.58 (13.32)*	17.05 (12.36)*	16.56 (12.03)*	15.76 (11.78)*	18.25 (14.05)*	27.61 (14.82)*	50.39 (24.22)**
	Model 2	Intercept	939.63 (71.87)***	1,247.75 (63.40)***	1,831.38 (62.78)***	2,628.92 (68.23)***	3,655.96 (80.41)***	4,732.88 (77.46)***	5,568.71 (111.89)***
		Cycle	68.54 (47.97)*	71.38 (42.31)*	66.85 (41.9)*	40.43 (45.53)*	-16.46 (53.66)	-34.02 (51.70)	-81.32 (74.67)*
		Cycle^2	-6.99 (6.59)*	-7.76 (5.81)*	-7.18 (5.76)*	-3.52 (6.25)	4.96 (7.37)	8.80 (7.10)*	18.82 (10.26)*
Hypertension (Without)[c]	Model 1	Intercept	1,142.5 (63.57)***	1,523.92 (64.41)***	2,200.75 (52.52)***	3,165.17 (46.89)***	4,384.58 (52.33)***	5,860.67 (62.35)***	6,915.00 (62.34)***
		Cycle	21.39 (15.20)*	15.95 (15.40)*	15.04 (12.55)*	12.52 (11.21)*	9.51 (12.51)*	20.13 (14.90)*	35.68 (14.90)***
	Model 2	Intercept	1,043.71 (59.18)***	1,422.67 (59.04)***	2,131.29 (57.29)***	3,135.83 (63.78)***	4,329.54 (64.14)***	5,907.04 (82.98)***	6,955.13 (84.53)***
		Cycle	120.18 (39.50)***	117.20 (39.40)***	84.49 (38.24)**	41.86 (42.56)*	64.55 (42.81)*	-26.24 (55.38)	-4.45 (56.41)
		Cycle^2	-14.11 (5.43)***	-14.46 (5.41)***	-9.92 (5.25)*	-4.19 (5.85)	-7.86 (5.88)*	6.62 (7.61)*	5.73 (7.75)*
Hypertension (With)[c]	Model 1	Intercept	1,043.00 (64.92)***	1,356.08 (51.79)***	1,936.67 (65.84)***	2,775.08 (53.00)***	3,868.75 (62.74)***	5,127.58 (61.31)***	6,180.42 (63.37)***
		Cycle	24.93 (15.52)*	24.40 (12.38)**	33.13 (15.74)**	36.76 (12.67)***	51.75 (15.00)***	62.65 (14.66)***	65.24 (15.15)***
	Model 2	Intercept	969.50 (77.28)***	1,309.83 (66.57)***	1,856.71 (75.74)***	2,729.08 (68.58)***	3,850.79 (88.74)***	5,144.63 (86.76)***	6,212.08 (87.78)***
		Cycle	98.43 (51.58)*	70.65 (44.43)*	113.09 (50.55)**	82.76 (45.77)*	69.71 (59.22)*	45.61 (57.90)*	33.57 (58.58)
		Cycle^2	-10.50 (7.08)*	-6.61 (6.10)*	-11.42 (6.94)*	-6.57 (6.29)*	-2.57 (8.13)	2.43 (7.95)	4.52 (8.05)
Heart disease (Without)[c]	Model 1	Intercept	1,118.00 (60.55)***	1,469.58 (55.61)***	2,125.17 (56.50)***	3,049.75 (53.76)***	4,246.67 (54.62)***	5,684.58 (55.50)***	6,738.83 (55.10)***
		Cycle	20.21 (14.47)*	17.44 (13.29)*	17.92 (13.51)*	19.04 (12.85)*	17.95 (13.06)*	28.58 (13.27)**	39.15 (13.17)***
	Model 2	Intercept	1,038.83 (66.59)***	1,392.13 (58.27)***	2,054.79 (64.09)***	3,022.13 (74.33)***	4,221.25 (75.97)***	5,731.88 (72.09)***	6,736.13 (78.68)***
		Cycle	99.38 (44.44)**	94.90 (38.89)**	88.29 (42.77)**	46.66 (49.60)*	43.37 (50.70)*	-18.71 (48.11)	41.86 (52.51)*
		Cycle^2	-11.31 (6.10)*	-11.07 (5.34)**	-10.05 (5.88)*	-3.95 (6.81)	-3.63 (6.96)	6.76 (6.61)*	-0.39 (7.21)
Heart disease (With)[c]	Model 1	Intercept	1,025.25 (119.49)***	1,366.5 (101.43)***	1,884.75 (82.83)***	2,608.92 (57.82)***	3,551.25 (59.17)***	4,726.67 (93.42)***	5,603.58 (174.7)***
		Cycle	35.07 (28.56)*	21.11 (24.25)*	36.71 (19.80)*	56.85 (13.82)***	84.07 (14.14)***	69.77 (22.33)***	61.37 (41.76)*

	Model 2	Intercept	921.25 (154.50)***	1,264.13 (126.09)***	1,811.83 (106.84)***	2,519.63 (54.28)***	3,556.67 (84.42)***	4,826.21 (113.98)***	5,770.13 (220.89)***
		Cycle	139.07 (103.11)*	123.48 (84.15)*	109.63 (71.30)*	146.14 (36.23)***	78.65 (56.34)*	-29.77 (76.06)	-105.17 (147.41)
		Cycle²	-14.86 (14.16)*	-14.63 (11.56)*	-10.42 (9.79)*	-12.76 (4.98)**	0.77 (7.74)	14.22 (10.45)*	23.79 (20.25)*
Heart attack (Without)[c]	Model 1	Intercept	1,125.42 (57.56)***	1,479.33 (53.15)***	2,130.17 (54.99)***	3,054.08 (49.47)***	4,250.00 (52.79)***	5,689.08 (56.43)***	6,742.58 (54.86)***
		Cycle	20.02 (13.76)*	16.73 (12.71)*	17.35 (13.15)*	18.73 (11.82)*	17.46 (12.62)*	25.48 (13.49)*	37.30 (13.11)***
	Model 2	Intercept	1,041.17 (57.54)***	1,398.04 (50.54)***	2,058.71 (60.74)***	3,024.04 (67.48)***	4,218.04 (72.03)***	5,736.25 (73.59)***	6,745.21 (78.33)***
		Cycle	104.27 (38.40)***	98.02 (33.73)***	88.80 (40.53)**	48.77 (45.03)*	49.42 (48.07)*	-21.69 (49.11)	34.67 (52.28)
		Cycle²	-12.04 (5.28)**	-11.61 (4.63)**	-10.21 (5.57)*	-4.29 (6.19)	-4.57 (6.60)	6.74 (6.75)*	0.37 (7.18)
Heart attack (With)[c]	Model 1	Intercept	986.75 (113.80)***	1,250.00 (104.05)***	1,729.75 (103.22)***	2,577.58 (90.58)***	3,503.5 (125.03)***	4,664.92 (164.12)***	5,552.17 (155.16)***
		Cycle	12.21 (27.2)	24.46 (24.87)*	54.96 (24.67)**	42.15 (21.65)**	90.04 (29.89)***	116.67 (39.23)***	122.67 (37.09)***
	Model 2	Intercept	1,025.42 (160.29)***	1,236.29 (148.31)***	1,671.46 (141.71)***	2,566.13 (129.13)***	3,634.63 (153.43)***	4,858.00 (191.94)***	5,650.08 (210.84)***
		Cycle	-26.45 (106.97)	38.17 (98.98)	113.26 (94.58)*	53.61 (86.18)	-41.09 (102.39)	-76.42 (128.10)	24.75 (140.71)
		Cycle²	5.52 (14.69)	-1.96 (13.60)	-8.33 (12.99)	-1.64 (11.84)	18.73 (14.06)*	27.58 (17.60)*	13.99 (19.33)*
Stroke (Without)[c]	Model 1	Intercept	1,126.42 (56.89)***	1,475.33 (53.22)***	2,130.08 (57.40)***	3,061.25 (47.87)***	4,246.83 (55.28)***	5,690.67 (57.58)***	6,749.00 (55.38)***
		Cycle	20.42 (13.60)*	18.76 (12.72)*	18.12 (13.72)*	19.25 (11.44)*	19.33 (13.21)*	26.92 (13.77)**	35.96 (13.24)***
	Model 2	Intercept	1,047.54 (59.83)***	1,401.33 (55.84)***	2,058.17 (64.88)***	3,035.46 (65.97)***	4,217.08 (76.19)***	5,742.71 (73.82)***	6,745.96 (79.06)***
		Cycle	99.29 (39.93)**	92.76 (37.27)**	90.04 (43.30)**	45.04 (44.02)*	49.08 (50.84)*	-25.12 (49.26)	39.01 (52.76)*
		Cycle²	-11.27 (5.48)**	-10.57 (5.12)**	-10.27 (5.95)*	-3.68 (6.05)	-4.25 (6.98)	7.43 (6.77)*	-0.43 (7.25)
Stroke (With)[c]	Model 1	Intercept	878.83 (106.33)***	1,159.67 (105.49)***	1,763.92 (83.25)***	2,438.17 (56.41)***	3,264.83 (85.31)***	4,492.50 (74.71)***	4,988.00 (186.62)***
		Cycle	26.80 (25.42)*	21.81 (25.22)*	12.45 (19.90)	40.06 (13.48)***	73.73 (20.39)***	119.86 (17.86)***	213.50 (44.61)***
	Model 2	Intercept	837.96 (149.17)***	1,044.42 (127.47)***	1,646.58 (86.32)***	2,380.54 (69.85)***	3,300.79 (119.24)***	4,559.00 (96.11)***	5048.42 (263.20)***
		Cycle	67.67 (99.55)	137.06 (85.07)*	129.79 (57.61)**	97.68 (46.61)**	37.77 (79.58)	53.36 (64.14)*	153.08 (175.65)*
		Cycle²	-5.84 (13.67)	-16.46 (11.68)*	-16.76 (7.91)**	-8.23 (6.40)*	5.14 (10.93)	9.50 (8.81)*	8.63 (24.13)
Having no condition	Model 1	Intercept	1,155.08 (65.95)***	1,535.92 (65.37)***	2,219.58 (52.11)***	3,184.75 (46.27)***	4,425.5 (47.52)***	5,894.42 (64.28)***	6,939.42 (58.71)***
		Cycle	19.83 (15.77)*	16.10 (15.63)*	13.15 (12.46)*	11.46 (11.06)*	4.29 (11.36)	18.35 (15.36)*	35.60 (14.04)***
	Model 2	Intercept	1,050.67 (59.82)***	1,432.75 (59.57)***	2,151.38 (57.27)***	3,154.29 (62.58)***	4,375.25 (58.14)***	5,952.54 (82.38)***	6,981.00 (78.69)***
		Cycle	124.25 (39.92)***	119.26 (39.75)***	81.36 (38.22)**	41.92 (41.76)*	54.54 (38.80)*	-39.78 (54.98)	-5.99 (52.52)
		Cycle²	-14.92 (5.48)***	-14.74 (5.46)***	-9.74 (5.25)*	-4.35 (5.74)*	-7.18 (5.33)*	8.30 (7.55)*	5.94 (7.21)*
	Model 1	Intercept	1,038.25	1,354.75	1,936.33	2,762.92	3,843.00	5,105.67	6,157.58

Having >=1 condition			(64.32)***	(50.85)***	(65.71)***	(48.68)***	(58.72)***	(64.69)***	(67.53)***
		Cycle	25.29 (15.38)*	23.18 (12.16)*	33.08 (15.71)**	37.99 (11.64)***	54.21 (14.04)***	62.95 (15.47)***	66.01 (16.14)***
	Model 2	Intercept	969.17 (78.24)***	1,308.79 (65.19)***	1,857.71 (76.20)***	2,720.79 (63.03)***	3,829.25 (83.32)***	5,128.00 (91.08)***	6176.04 (95.59)***
		Cycle	94.37 (52.22)*	69.14 (43.50)*	111.71 (50.86)**	80.11 (42.07)*	67.96 (55.61)*	40.62 (60.79)	47.55 (63.80)*
		$Cycle^2$	-9.87 (7.17)*	-6.57 (5.98)*	-11.23 (6.99)*	-6.02 (5.78)*	-1.96 (7.64)	3.19 (8.35)	2.64 (8.76)

*P-value < 0.5; **P-value<0.1; ***P-value<0.05.

a. P represents percentiles (P5, P10, P25, P50, P75, P90, and P95).
b. β = beta coefficients, SE= standard error.
c. “With” and “Without” indicates participants’ health conditions.

Table S5. Sensitivity analysis: Descriptive summary of sodium intake among children (Ages 5–17) and adults (Ages 18 and older) stratified by sex and in adults based on health conditions, pooled across eight NHANES cycles (2003–2018), based on weighted data

Group [a]	N [b]	Mean	SD[c]	Range	Min[d]	P05[e]	P10[e]	P25[e]	P50[e]	P75[e]	P90[e]	P95[e]	Max[f]	Skew[g]	Kurt[h]
Children (5-17)	18,194	3,178	3,115	20,325	0	1,236	1,544	2,110	2,873	3,879	5,160	6,122	20,325	1.62	5.50
Adults (18 and older)	42,469	3,535	2,868	25,949	0	1,250	1,612	2,278	3,211	4,399	5,837	6,880	25,949	1.64	5.87
Boys	9,198	3,486	2,964	20,325	0	1,384	1,692	2,305	3,133	4,235	5,696	6,856	20,325	1.60	5.22
Male	20,637	4,144	2,989	25,949	0	1,548	1,979	2,744	3,839	5,123	6,667	7,849	25,949	1.47	4.70
Girls	8,996	2,862	2,487	15,976	0	1,151	1,432	1,957	2,631	3,503	4,522	5,325	15,976	1.42	4.33
Female	21,832	2,967	1,996	21,004	0	1,101	1,418	2,001	2,770	3,688	4,711	5,495	21,004	1.62	7.35
Hypertension (Without)[g]	28,095	3,605	2,597	25,949	0	1,267	1,646	2,338	3,283	4,470	5,952	6,991	25,949	1.64	5.92
Hypertension (With)[g]	14,374	3,378	2,976	20,999	5	1,223	1,553	2,154	3,060	4,241	5,565	6,656	21,004	1.60	5.48
Heart disease (Without)[g]	40,792	3,547	2,816	25,949	0	1,255	1,616	2,285	3,222	4,418	5,854	6,897	25,949	1.64	5.88
Heart disease (With)[g]	1,677	3,209	2,261	11,474	5	1,144	1,524	2,084	2,950	3,961	5,349	6,346	11,479	1.10	1.98
Heart attack (Without)[g]	40,716	3,545	2,858	25,949	0	1,264	1,621	2,286	3,220	4,413	5,849	6,889	25,949	1.64	5.87
Heart attack (With)[g]	1,753	3,254	3,072	15,720	5	1,046	1,384	2,042	2,908	4,004	5,466	6,576	15,725	1.65	5.79
Stroke (Without)[g]	40,913	3,553	2,794	25,949	0	1,264	1,624	2,293	3,230	4,421	5,865	6,905	25,949	1.64	5.90
Stroke (With)[g]	1,556	2,908	2,237	12,786	75	951	1,306	1,854	2,646	3,613	4,782	5,665	12,861	1.38	3.12
Having no condition	27,113	3,618	2,539	25,949	0	1,272	1,649	2,346	3,296	4,493	5,961	7,008	25,949	1.64	5.90
Having >=1 condition	15,356	3,366	2,930	20,999	5	1,217	1,548	2,154	3,053	4,218	5,536	6,627	21,004	1.61	5.54

a. "With" and "Without" indicates participants’ health condition.
b. N = sample size.
c. SD = standard deviation.
d. Min = minimum.
e. P represents percentiles (P5, P10, P25, P50, P75, P90, and P95).
f. Max = maximum.
g. Skew = skewness, calculated based on unweighted data.
h. Kurt = kurtosis, calculated based on unweighted data.

Table S6. Sensitivity analysis: Descriptive summary of sodium intake among children (Ages 5–17) and adults (Ages 18 and older) stratified by sex and in adults based on health conditions, by NHANES Cycle (2003–2018), based on weighted data

Group [a]	Yr[b]	N [c]	Mean	SD[d]	Range	Min[e]	P05[f]	P10[f]	P25[f]	P50[f]	P75[f]	P90[f]	P95[f]	Max[g]	Skew[h]	Kurt[i]

Children (5-17)																
	2003	2,698	3,308	2,337	12,131	0	1,377	1,602	2,227	2,991	4,088	5,372	6,406	12,131	1.23	2.32
	2005	2,778	3,306	3,250	15,503	195	1,217	1,577	2,146	3,026	3,934	5,207	6,254	15,698	1.91	6.89
	2007	2,175	3,131	3,544	20,218	107	1,206	1,503	2,019	2,777	3,810	5,170	6,214	20,325	1.87	9.18
	2009	2,306	3,200	3,512	15,713	263	1,283	1,595	2,148	2,890	3,892	5,185	6,127	15,976	1.74	6.16
	2011	2,187	3,184	3,828	14,150	115	1,178	1,571	2,208	2,938	3,885	5,170	6,016	14,265	1.33	3.40
	2013	2,186	3,118	2,442	17,475	17	1,224	1,557	2,053	2,840	3,821	5,023	5,690	17,492	1.64	5.93
	2015	2,135	3,152	2,006	16,414	0	1,265	1,529	2,113	2,833	3,808	5,067	6,141	16,414	1.66	5.55
	2017	1,729	3,031	3,023	11,554	42	1,169	1,398	2,005	2,714	3,836	4,942	5,963	11,596	1.34	2.99
Adults (18 and older)																
	2003	4,986	3,486	2,302	15,047	15	1,136	1,512	2,197	3,198	4,347	5,930	6,853	15,062	1.30	2.80
	2005	5,060	3,535	3,711	18,022	31	1,220	1,625	2,245	3,190	4,475	5,800	6,799	18,053	1.52	4.65
	2007	5,690	3,443	4,319	21,248	0	1,136	1,487	2,159	3,090	4,346	5,759	6,833	21,248	1.85	7.64
	2009	6,052	3,594	2,206	17,613	25	1,396	1,736	2,341	3,237	4,437	5,842	7,017	17,638	1.53	4.31
	2011	5,076	3,618	1,869	20,176	7	1,401	1,706	2,352	3,271	4,517	5,837	6,876	20,183	1.62	5.33
	2013	5,356	3,534	2,262	21,370	29	1,291	1,644	2,346	3,239	4,377	5,766	6,746	21,399	1.80	7.25
	2015	5,266	3,535	2,983	16,536	34	1,292	1,653	2,299	3,246	4,371	5,787	6,827	16,570	1.42	3.73
	2017	4,983	3,530	2,769	25,949	0	1,185	1,551	2,246	3,203	4,342	5,943	7,040	25,949	1.99	9.81
Boys																
	2003	1,354	3,549	2,909	12,131	0	1,456	1,762	2,375	3,191	4,384	5,843	6,873	12,131	1.15	2.05
	2005	1,379	3,677	3,021	15,503	195	1,565	1,786	2,423	3,269	4,244	5,799	7,307	15,698	1.91	6.21
	2007	1,113	3,471	2,773	20,218	107	1,333	1,639	2,220	2,953	4,261	5,883	7,019	20,325	1.97	10.18
	2009	1,186	3,501	2,963	14,523	442	1,387	1,705	2,367	3,127	4,161	5,652	7,151	14,965	1.49	3.73
	2011	1,117	3,479	3,698	14,018	247	1,374	1,723	2,383	3,162	4,184	5,654	6,904	14,265	1.31	3.39
	2013	1,119	3,476	2,973	17,318	174	1,409	1,713	2,288	3,185	4,367	5,396	6,476	17,492	1.68	5.92
	2015	1,077	3,421	2,383	16,146	268	1,356	1,622	2,257	3,036	4,060	5,723	6,824	16,414	1.59	4.92
	2017	853	3,315	2,607	11,447	149	1,319	1,624	2,161	3,008	4,186	5,596	6,166	11,596	1.26	2.62
Male																
	2003	2,395	4,104	2,505	15,027	35	1,449	1,857	2,690	3,848	5,092	6,698	7,578	15,062	1.14	2.03
	2005	2,425	4,182	3,200	17,924	129	1,538	1,961	2,715	3,869	5,230	6,752	7,937	18,053	1.37	3.36
	2007	2,809	4,050	4,421	21,248	0	1,487	1,911	2,630	3,734	5,006	6,536	7,855	21,248	1.66	6.17
	2009	2,946	4,255	2,364	17,454	184	1,718	2,076	2,803	3,925	5,214	6,829	8,252	17,638	1.34	3.18
	2011	2,535	4,255	2,210	15,178	7	1,679	2,067	2,888	3,985	5,251	6,670	7,689	15,185	1.32	2.95
	2013	2,558	4,097	2,550	21,370	29	1,602	2,039	2,772	3,795	4,966	6,469	7,854	21,399	1.61	5.45
	2015	2,543	4,095	3,293	15,908	82	1,592	2,032	2,736	3,773	5,001	6,521	7,614	15,990	1.25	2.76
	2017	2,426	4,115	2,921	25,927	22	1,415	1,837	2,687	3,784	5,141	6,835	7,949	25,949	1.99	9.95
Girls																
	2003	1,344	3,047	2,242	10,141	192	1,308	1,503	2,040	2,826	3,714	4,827	5,764	10,333	1.24	2.31
	2005	1,399	2,915	2,528	11,436	269	1,132	1,364	1,924	2,706	3,689	4,568	5,423	11,705	1.39	4.11
	2007	1,062	2,801	3,093	10,783	149	1,119	1,416	1,843	2,549	3,377	4,326	5,167	10,932	1.44	4.11
	2009	1,120	2,909	2,660	15,713	263	1,217	1,538	2,038	2,656	3,515	4,715	5,623	15,976	2.04	11.81
	2011	1,070	2,892	2,389	9,053	115	1,071	1,413	2,084	2,738	3,549	4,469	5,267	9,168	1.21	2.63
	2013	1,067	2,726	1,478	8,777	17	1,101	1,407	1,878	2,621	3,264	4,212	4,944	8,794	1.09	1.94
	2015	1,058	2,867	1,528	13,096	0	1,177	1,446	2,029	2,586	3,446	4,438	5,373	13,096	1.60	5.61
	2017	876	2,741	2,838	9,778	42	1,151	1,328	1,854	2,468	3,424	4,454	4,982	9,820	1.25	2.56
Female																
	2003	2,591	2,913	1,940	11,731	15	984	1,330	1,904	2,699	3,687	4,656	5,581	11,746	1.17	2.51
	2005	2,635	2,935	2,510	17,394	31	1,063	1,397	1,988	2,757	3,659	4,776	5,455	17,425	1.21	4.93
	2007	2,881	2,898	2,169	18,561	5	983	1,267	1,868	2,668	3,563	4,697	5,488	18,566	2.02	10.97
	2009	3,106	2,976	1,553	13,675	25	1,235	1,539	2,089	2,778	3,680	4,692	5,329	13,700	1.23	3.61
	2011	2,541	3,002	1,799	20,050	133	1,173	1,547	2,067	2,828	3,688	4,697	5,389	20,183	2.14	12.72
	2013	2,798	3,002	1,310	20,959	45	1,150	1,423	2,035	2,806	3,689	4,728	5,582	21,004	1.86	10.92
	2015	2,723	3,013	1,976	16,536	34	1,103	1,452	2,040	2,759	3,764	4,734	5,564	16,570	1.48	5.11
	2017	2,557	2,986	2,321	14,473	0	1,130	1,392	1,964	2,808	3,726	4,732	5,529	14,473	1.59	5.22
Hypertension (Without)																
	2003	3,404	3,583	2,580	14,049	15	1,172	1,572	2,290	3,325	4,444	6,070	7,077	14,064	1.23	2.44
	2005	3,599	3,638	3,460	18,022	31	1,244	1,632	2,302	3,305	4,574	5,975	7,072	18,053	1.50	4.40
	2007	3,734	3,545	3,437	21,248	0	1,150	1,514	2,242	3,195	4,434	5,963	6,922	21,248	1.90	8.21
	2009	3,998	3,645	2,253	14,974	25	1,421	1,759	2,388	3,290	4,516	5,873	7,123	14,999	1.44	3.70
	2011	3,328	3,662	2,318	16,964	106	1,398	1,763	2,402	3,308	4,590	5,949	6,948	17,070	1.52	4.28
	2013	3,471	3,602	2,018	21,354	45	1,350	1,710	2,394	3,311	4,451	5,854	6,781	21,399	1.75	6.68
	2015	3,407	3,597	2,254	16,536	34	1,254	1,683	2,339	3,317	4,458	5,880	6,912	16,570	1.47	4.11
	2017	3,154	3,569	2,381	25,949	0	1,183	1,539	2,314	3,245	4,341	6,013	7,040	25,949	2.18	11.39

Hypertension (With)															
	2003	1,582	3,260	2,302	14,934	128	1,110	1,397	2,000	2,990	4,180	5,528	6,451	15,062	1.45	3.88
	2005	1,461	3,292	3,466	17,340	85	1,179	1,607	2,130	2,975	4,265	5,432	6,345	17,425	1.53	5.31
	2007	1,956	3,202	3,460	15,189	5	1,106	1,412	2,015	2,843	4,042	5,317	6,529	15,194	1.64	5.01
	2009	2,054	3,470	3,039	17,431	207	1,364	1,698	2,269	3,087	4,197	5,721	6,883	17,638	1.73	5.90
	2011	1,748	3,518	2,321	20,176	7	1,400	1,633	2,268	3,185	4,423	5,615	6,670	20,183	1.85	8.14
	2013	1,885	3,402	2,123	20,975	29	1,201	1,509	2,212	3,090	4,202	5,510	6,694	21,004	1.91	8.69
	2015	1,859	3,397	4,165	11,836	82	1,374	1,645	2,231	3,109	4,218	5,550	6,557	11,918	1.26	2.38
	2017	1,829	3,447	2,436	14,206	9	1,221	1,561	2,100	3,144	4,342	5,782	7,030	14,215	1.37	3.46
Heart disease (Without)															
	2003	4,753	3,502	2,185	15,047	15	1,136	1,514	2,206	3,211	4,367	5,958	6,864	15,062	1.30	2.79
	2005	4,883	3,551	3,756	18,022	31	1,227	1,631	2,252	3,206	4,490	5,837	6,832	18,053	1.52	4.61
	2007	5,467	3,454	4,166	21,248	0	1,132	1,485	2,165	3,095	4,361	5,786	6,845	21,248	1.86	7.65
	2009	5,817	3,602	2,158	17,613	25	1,397	1,737	2,348	3,242	4,447	5,851	7,013	17,638	1.52	4.30
	2011	4,904	3,625	1,842	20,176	7	1,401	1,711	2,362	3,287	4,531	5,845	6,900	20,183	1.62	5.31
	2013	5,153	3,551	2,279	21,354	45	1,299	1,650	2,356	3,254	4,390	5,792	6,748	21,399	1.81	7.33
	2015	5,053	3,545	2,839	16,536	34	1,286	1,653	2,300	3,263	4,384	5,789	6,858	16,570	1.42	3.72
	2017	4,762	3,537	2,771	25,949	0	1,199	1,562	2,248	3,208	4,348	5,957	7,022	25,949	2.02	9.93
Heart disease (With)															
	2003	233	3,132	1,995	7,860	484	1,089	1,436	1,953	3,005	3,860	4,893	6,068	8,344	1.10	1.21
	2005	177	3,020	2,115	7,410	349	1,020	1,244	2,035	2,798	3,741	5,321	5,590	7,759	0.69	0.61
	2007	223	3,130	2,151	11,474	5	1,280	1,521	1,917	2,831	3,915	4,854	6,092	11,479	1.40	3.92
	2009	235	3,324	2,015	8,117	515	1,320	1,707	2,099	2,984	4,064	5,231	7,023	8,632	1.15	1.74
	2011	172	3,364	2,420	10,943	251	1,336	1,620	2,257	2,928	3,906	5,473	6,616	11,194	1.63	4.78
	2013	203	3,078	1,424	9,110	29	956	1,386	1,953	2,842	3,944	4,907	5,996	9,139	1.03	1.46
	2015	213	3,259	1,998	8,009	763	1,508	1,654	2,205	3,096	3,855	5,387	6,494	8,772	0.93	0.92
	2017	221	3,359	3,012	8,404	75	1,067	1,236	2,084	3,067	4,229	5,902	7,033	8,479	0.72	0.36
Heart attack (Without)															
	2003	4,743	3,500	2,119	15,047	15	1,135	1,513	2,199	3,208	4,367	5,958	6,866	15,062	1.29	2.74
	2005	4,870	3,550	3,770	18,022	31	1,227	1,632	2,252	3,201	4,498	5,835	6,827	18,053	1.52	4.65
	2007	5,435	3,464	4,092	21,248	0	1,149	1,498	2,176	3,107	4,368	5,788	6,846	21,248	1.87	7.72
	2009	5,810	3,597	2,203	17,613	25	1,414	1,740	2,349	3,241	4,446	5,843	6,959	17,638	1.50	4.16
	2011	4,900	3,633	1,847	20,176	7	1,402	1,713	2,369	3,291	4,541	5,861	6,938	20,183	1.61	5.24
	2013	5,154	3,547	2,541	21,354	45	1,291	1,648	2,353	3,254	4,390	5,782	6,749	21,399	1.78	7.15
	2015	5,048	3,531	2,964	16,536	34	1,298	1,656	2,300	3,249	4,372	5,784	6,813	16,570	1.43	3.78
	2017	4,756	3,531	2,818	25,949	0	1,199	1,562	2,251	3,204	4,336	5,933	7,007	25,949	2.03	10.00
Heart attack (With)															
	2003	243	3,191	2,689	11,533	483	1,151	1,483	1,959	3,078	3,904	4,978	6,225	12,016	1.51	4.41
	2005	190	3,118	1,859	8,869	509	1,086	1,310	1,963	2,999	3,868	5,327	5,751	9,378	1.19	2.12
	2007	255	2,827	2,366	11,474	5	685	1,142	1,778	2,417	3,720	4,634	5,925	11,479	1.40	3.83
	2009	242	3,508	2,906	13,314	650	971	1,602	2,075	3,088	4,235	5,646	7,642	13,964	2.12	8.07
	2011	176	3,098	1,358	13,298	251	955	1,533	2,163	2,816	3,778	5,105	5,692	13,549	2.13	9.62
	2013	202	3,123	2,489	15,696	29	1,217	1,505	2,115	2,709	3,723	4,769	6,124	15,725	2.37	10.79
	2015	218	3,639	4,608	10,220	505	1,048	1,481	2,239	3,130	4,239	6,254	7,812	10,725	1.15	1.70
	2017	227	3,498	3,910	8,171	175	982	1,210	2,111	3,173	4,551	6,335	7,192	8,346	0.71	0.03
Stroke (Without)															
	2003	4,806	3,507	2,251	15,047	15	1,160	1,526	2,211	3,229	4,362	5,959	6,904	15,062	1.30	2.77
	2005	4,891	3,559	3,604	18,022	31	1,227	1,632	2,255	3,213	4,506	5,845	6,829	18,053	1.52	4.66
	2007	5,458	3,467	4,085	21,248	0	1,144	1,495	2,181	3,112	4,370	5,795	6,846	21,248	1.86	7.67
	2009	5,849	3,610	2,075	17,613	25	1,414	1,744	2,359	3,249	4,452	5,854	7,087	17,638	1.51	4.24
	2011	4,887	3,637	1,875	20,176	7	1,400	1,711	2,369	3,297	4,542	5,865	6,935	20,183	1.62	5.31
	2013	5,186	3,554	2,340	21,370	29	1,302	1,651	2,366	3,256	4,406	5,787	6,750	21,399	1.81	7.37
	2015	5,082	3,546	2,989	16,536	34	1,302	1,669	2,300	3,247	4,376	5,801	6,861	16,570	1.43	3.76
	2017	4,754	3,543	2,695	25,949	0	1,200	1,566	2,265	3,212	4,353	5,951	7,014	25,949	2.02	10.04
Stroke (With)															
	2003	180	2,757	2,351	8,599	332	640	965	1,651	2,461	3,706	4,755	5,099	8,931	1.09	1.83
	2005	169	2,685	1,701	7,719	424	861	1,255	1,813	2,439	3,320	4,513	4,778	8,143	1.12	1.69
	2007	232	2,738	1,789	9,594	115	753	1,289	1,807	2,578	3,260	4,616	5,447	9,709	1.26	2.63
	2009	203	2,955	1,783	12,285	576	1,082	1,486	1,953	2,623	3,536	4,653	5,740	12,861	2.01	7.75
	2011	189	2,932	1,708	8,170	707	1,401	1,583	2,001	2,632	3,343	4,831	5,357	8,877	1.21	1.98
	2013	170	2,812	1,956	10,498	381	896	1,260	1,841	2,598	3,341	4,388	5,495	10,879	1.69	3.85
	2015	184	3,129	1,224	8,614	82	1,106	1,312	2,187	3,021	3,919	4,709	5,724	8,696	0.94	1.07
	2017	229	3,170	3,471	9,176	75	974	1,265	1,690	2,760	4,004	5,560	7,408	9,251	1.21	1.27

Having no condition																
	2,003	3,257	3,593	2,301	14,049	15	1,176	1,577	2,294	3,344	4,456	6,101	7,071	14,064	1.21	2.35
	2005	3,473	3,655	3,420	18,022	31	1,253	1,633	2,325	3,331	4,601	5,983	7,127	18,053	1.49	4.39
	2007	3,585	3,564	3,284	21,248	0	1,157	1,525	2,273	3,212	4,452	5,984	6,949	21,248	1.90	8.24
	2009	3,875	3,657	2,261	14,974	25	1,427	1,762	2,390	3,306	4,552	5,877	7,122	14,999	1.44	3.69
	2011	3,239	3,666	2,263	16,964	106	1,400	1,756	2,404	3,308	4,591	5,947	6,950	17,070	1.52	4.26
	2013	3,364	3,619	1,984	21,354	45	1,355	1,736	2,406	3,334	4,465	5,882	6,813	21,399	1.72	6.49
	2015	3,284	3,610	2,368	16,536	34	1,273	1,698	2,341	3,325	4,473	5,907	6,941	16,570	1.47	4.08
	2017	3,036	3,581	2,330	25,949	0	1,182	1,539	2,316	3,251	4,362	6,095	7,085	25,949	2.20	11.43
Having >=1 condition																
	2003	1,729	3,265	2,268	14,934	128	1,104	1,417	2,000	2,995	4,178	5,513	6,459	15,062	1.48	4.04
	2005	1,587	3,278	3,434	17,340	85	1,172	1,586	2,132	2,972	4,240	5,378	6,322	17,425	1.52	5.23
	2007	2,105	3,184	3,628	15,189	5	1,111	1,406	2,006	2,837	4,015	5,294	6,466	15,194	1.65	5.07
	2009	2,177	3,453	3,009	17,431	207	1,357	1,685	2,265	3,063	4,179	5,697	6,888	17,638	1.72	5.86
	2011	1,837	3,518	2,103	20,176	7	1,400	1,636	2,269	3,190	4,404	5,656	6,619	20,183	1.85	8.03
	2013	1,992	3,382	1,940	20,975	29	1,200	1,514	2,202	3,069	4,189	5,479	6,655	21,004	1.97	9.01
	2015	1,982	3,383	4,216	11,836	82	1,335	1,623	2,231	3,108	4,212	5,533	6,530	11,918	1.25	2.40
	2017	1,947	3,432	2,277	14,206	9	1,230	1,560	2,113	3,140	4,285	5,733	6,889	14,215	1.35	3.35

a. "With" and "Without" indicates participants' health conditions.
b. Yr represents Cycle.
c. N = sample size.
d. SD = standard deviation.
e. Min = minimum.
f. P represents percentiles (P5, P10, P25, P50, P75, P90, and P95).
g. Max = maximum.
h. Skew = skewness, calculated based on unweighted data.
i. Kurt = kurtosis, calculated based on unweighted data.

Table S7. Sensitivity analysis: Temporal Trends in Sodium Intake, Based on Weighted Population-Level Data Among Children (Ages 5–17 Years) and Adults (Ages 18 and Older), Adjusted for Survey Cycle

			**P05[a]**	**P10[a]**	**P25[a]**	**P50[a]**	**P75[a]**	**P90[a]**	**P95[a]**
**Group[c]**	**Model**	**Term**	**β (SE)[b]**	**β (SE)[b]**	**β (SE)[b]**	**β (SE)[b]**	**β (SE)[b]**	**β (SE)[b]**	**β (SE)[b]**
Children (5-17)	Model 1	Intercept	1,292.50 (39.49)***	1,605.31 (34.66)***	2,179.61 (48.47)***	2,987.21 (50.53)***	3,982.84 (44.27)***	5,315.93 (34.50)***	6,324.15 (104.16)***
		Cycle	-15.06 (9.44)*	-18.24 (8.29)**	-18.53 (11.59)*	-31.75 (12.08)***	-28.21 (10.58)***	-49.65 (8.25)***	-63.66 (24.90)***
	Model 2	Intercept	1322.19 (52.48)***	1567.60 (41.95)***	2174.75 (69.14)***	2978.94 (71.93)***	4042.35 (47.72)***	5320.83 (49.16)***	6430.56 (128.96)***
		Cycle	-44.74 (35.02)*	19.47 (28.00)	-13.67 (46.14)	-23.49 (48.01)	-87.72 (31.85)***	-54.55 (32.81)*	-170.07 (86.06)*
		Cycle²	4.24 (4.81)*	-5.39 (3.85)*	-0.69 (6.34)	-1.18 (6.59)	8.50 (4.37)*	0.70 (4.51)	15.20 (11.82)*
Adults (18 and older)	Model 1	Intercept	1,208.17 (69.85)***	1,578.98 (60.09)***	2,223.46 (44.65)***	3,176.32 (35.10)***	4,417.66 (45.95)***	5,831.21 (49.20)***	6,830.21 (68.66)***
		Cycle	13.94 (16.70)*	10.13 (14.36)	14.19 (10.67)*	9.43 (8.39)*	-4.59 (10.98)	0.52 (11.76)	12.50 (16.41)*
	Model 2	Intercept	1,103.66 (68.17)***	1,500.45 (66.10)***	2,167.96 (50.71)***	3,164.31 (49.43)***	4,364.50 (54.18)***	5,903.14 (49.24)***	6,858.17 (96.11)***
		Cycle	118.45 (45.49)***	88.66 (44.12)*	69.69 (33.84)**	21.45 (32.99)	48.57 (36.16)*	-71.42 (32.86)**	-15.46 (64.14)
		Cycle²	-14.93 (6.25)**	-11.22 (6.06)*	-7.93 (4.65)*	-1.72 (4.53)	-7.59 (4.97)*	10.28 (4.51)**	3.99 (8.81)
Boys	Model 1	Intercept	1,474.43 (41.78)***	1,761.15 (30.25)***	2,397.37 (47.57)***	3,187.51 (66.23)***	4,312.81 (63.65)***	5,841.73 (81.70)***	7,225.15 (173.09)***

		Cycle	-21.29 (9.99)**	-18.34 (7.23)***	-25.13 (11.37)**	-20.36 (15.83)*	-23.38 (15.22)*	-42.44 (19.53)**	-110.00 (41.38)***
	Model 2	Intercept	1,487.76 (58.94)***	1,757.53 (43.14)***	2,362.46 (63.43)***	3,181.20 (94.50)***	4,341.51 (88.69)***	5,892.86 (111.12)***	7,001.74 (192.04)***
		Cycle	-34.62 (39.34)*	-14.72 (28.79)	9.77 (42.33)	-14.05 (63.06)	-52.08 (59.19)*	-93.57 (74.16)*	113.40 (128.16)*
		Cycle²	1.90 (5.40)	-0.52 (3.95)	-4.99 (5.81)*	-0.90 (8.66)	4.10 (8.13)	7.30 (10.19)	-31.91 (17.60)*
Male	Model 1	Intercept	1,545.99 (74.7)***	1,947.78 (65.12)***	2,715.27 (54.51)***	3,867.37 (57.23)***	5,149.50 (77.03)***	6,687.10 (98.11)***	7,823.88 (152.14)***
		Cycle	4.04 (17.86)	7.04 (15.57)	7.13 (13.03)	-8.09 (13.68)	-10.53 (18.41)	-6.63 (23.45)	4.88 (36.37)
	Model 2	Intercept	1,418.00 (58.52)***	1,834.84 (49.54)***	2,649.69 (63.04)***	3,822.63 (75.55)***	5,133.56 (109.45)***	6,749.25 (133.26)***	7,715.72 (203.81)***
		Cycle	132.03 (39.06)***	119.98 (33.06)***	72.70 (42.07)*	36.65 (50.42)*	5.40 (73.05)	-68.78 (88.93)*	113.04 (136.02)*
		Cycle²	-18.29 (5.36)***	-16.13 (4.54)***	-9.37 (5.78)*	-6.39 (6.93)*	-2.28 (10.03)	8.88 (12.22)*	-15.45 (18.68)*
Girls	Model 1	Intercept	1,204.25 (47.52)***	1,467.17 (43.60)***	1,987.33 (66.26)***	2,760.75 (54.59)***	3,645.17 (78.43)***	4,661.50 (114.78)***	5,599.08 (147.44)***
		Cycle	-12.79 (11.36)*	-11.51 (10.42)*	-7.45 (15.84)	-33.43 (13.05)***	-42.26 (18.75)**	-45.82 (27.44)*	-80.35 (35.25)**
	Model 2	Intercept	1,263.50 (53.87)***	1,442.29 (59.82)***	1,963.92 (93.22)***	2,755.33 (77.87)***	3,722.58 (98.18)***	4,763.13 (147.86)***	5,650.54 (207.51)***
		Cycle	-72.04 (35.95)*	13.36 (39.92)	15.96 (62.21)	-28.01 (51.97)	-119.68 (65.52)*	-147.45 (98.67)*	-131.8 (138.49)*
		Cycle²	8.46 (4.94)*	-3.55 (5.48)	-3.35 (8.55)	-0.77 (7.14)	11.06 (9.00)*	14.52 (13.55)*	7.35 (19.02)
Female	Model 1	Intercept	1,033.56 (51.96)***	1,368.86 (62.19)***	1,946.32 (49.43)***	2,711.55 (29.27)***	3,632.43 (32.41)***	4,696.84 (23.71)***	5,467.80 (64.37)***
		Cycle	19.77 (12.42)*	14.16 (14.87)*	13.76 (11.82)*	14.70 (7.00)**	14.19 (7.75)*	4.93 (5.67)*	6.22 (15.39)
	Model 2	Intercept	972.25 (60.68)***	1,302.52 (75.84)***	1,888.29 (57.87)***	2,695.27 (40.24)***	3,662.20 (41.38)***	4,697.08 (33.86)***	5,550.80 (71.47)***
		Cycle	81.08 (40.50)*	80.49 (50.62)*	71.78 (38.62)*	30.98 (26.85)*	-15.58 (27.62)	4.69 (22.60)	-76.77 (47.69)*
		Cycle²	-8.76 (5.56)*	-9.48 (6.95)*	-8.29 (5.31)*	-2.33 (3.69)	4.25 (3.79)*	0.03 (3.10)	11.86 (6.55)*
Hypertension (Without)	Model 1	Intercept	1,242.19 (72.21)***	1,620.83 (66.82)***	2,299.51 (36.29)***	3,292.21 (30.91)***	4,524.81 (51.40)***	5,994.05 (46.82)***	7,053.48 (71.71)***
		Cycle	8.34 (17.26)	7.33 (15.97)	9.80 (8.67)*	-1.50 (7.39)	-13.93 (12.29)*	-13.41 (11.19)*	-19.76 (17.14)*
	Model 2	Intercept	1,133.47 (69.97)***	1,529.74 (71.26)***	2,258.40 (43.18)***	3,296.50 (44.05)***	4,455.65 (55.37)***	6,072.29 (38.70)***	7,109.58 (94.67)***
		Cycle	117.07 (46.70)**	98.42 (47.55)**	50.92 (28.82)*	-5.79 (29.40)	55.24 (36.95)*	-91.65 (25.83)***	-75.86 (63.18)*
		Cycle²	-15.53 (6.41)**	-13.01 (6.53)*	-5.87 (3.96)*	0.61 (4.04)	-9.88 (5.08)*	11.18 (3.55)***	8.01 (8.68)*
Hypertension (With)	Model 1	Intercept	1,158.10 (71.54)***	1,492.65 (69.83)***	2,078.15 (66.17)***	2,945.17 (56.33)***	4,167.00 (72.31)***	5,438.6 (87.01)***	6,420.17 (112.90)***
		Cycle	24.68 (17.10)*	18.58 (16.69)*	21.4 (15.82)*	30.79 (13.47)**	19.07 (17.29)*	33.80 (20.80)*	64.19 (26.99)**
	Model 2	Intercept	1,080.06 (86.50)***	1,431.87 (90.30)***	1,982.06 (66.69)***	2,939.24 (80.35)***	4,179.80 (102.89)***	5,480.17 (120.86)***	6,413.39 (161.19)***
		Cycle	102.72 (57.73)*	79.36 (60.26)*	117.49 (44.5)***	36.71 (53.62)	6.27 (68.67)	-7.77 (80.66)	70.97 (107.57)
		Cycle²	-11.15	-8.68	-13.73	-0.85	1.83	5.94	-0.97

			(7.93)*	(8.28)*	(6.11)**	(7.37)	(9.43)	(11.08)	(14.78)
Heart disease (Without)	Model 1	Intercept	1,207.86 (68.95)***	1,579.92 (59.87)***	2,232.67 (46.10)***	3,188.11 (37.58)***	4,435.16 (45.59)***	5,861.47 (48.57)***	6,850.39 (62.09)***
		Cycle	14.77 (16.48)*	10.84 (14.31)*	13.36 (11.02)*	9.36 (8.98)*	-5.82 (10.90)	-2.78 (11.61)	9.91 (14.84)
	Model 2	Intercept	1,106.71 (68.77)***	1,503.90 (67.11)***	2,174.85 (52.08)***	3,176.35 (53.05)***	4,382.48 (53.79)***	5,937.20 (45.03)***	6,873.23 (87.24)***
		Cycle	115.93 (45.89)**	86.86 (44.79)*	71.17 (34.76)**	21.11 (35.40)	46.85 (35.90)*	-78.52 (30.05)***	-12.93 (58.22)
		Cycle²	-14.45 (6.30)**	-10.86 (6.15)*	-8.26 (4.77)*	-1.68 (4.86)	-7.53 (4.93)*	10.82 (4.13)***	3.26 (8.00)
Heart disease (With)	Model 1	Intercept	1,141.80 (130.62)***	1,469.03 (126.18)***	1,977.93 (75.12)***	2,864.76 (67.97)***	3,810.70 (82.37)***	4,921.52 (193.55)***	5,923.03 (287.79)***
		Cycle	15.76 (31.22)	1.85 (30.16)	24.26 (17.96)*	22.63 (16.25)*	36.75 (19.69)*	92.69 (46.27)**	126.00 (68.8)*
	Model 2	Intercept	1,043.15 (173.43)***	1,312.15 (143.26)***	1,940.75 (104.12)***	2,940.29 (81.56)***	3,843.81 (115.35)***	5,066.22 (257.40)***	5,895.26 (410.59)***
		Cycle	114.41 (115.74)*	158.74 (95.61)*	61.43 (69.49)*	-52.91 (54.43)*	3.63 (76.98)	-52.01 (171.78)	153.77 (274.02)
		Cycle²	-14.09 (15.90)*	-22.41 (13.13)*	-5.31 (9.54)	10.79 (7.48)*	4.73 (10.57)	20.67 (23.6)*	-3.97 (37.64)
Heart attack (Without)	Model 1	Intercept	1,213.66 (69.77)***	1,583.21 (58.99)***	2,232.86 (45.07)***	3,190.3 (35.35)***	4,443.43 (48.57)***	5,865.6 (46.09)***	6,850.47 (58.42)***
		Cycle	14.50 (16.68)*	10.55 (14.10)*	13.77 (10.77)*	8.26 (8.45)*	-8.21 (11.61)	-5.04 (11.02)	7.22 (13.96)
	Model 2	Intercept	1,108.03 (67.16)***	1,504.70 (64.07)***	2,171.52 (48.13)***	3,173.35 (49.08)***	4,384.34 (55.82)***	5,933.33 (45.88)***	6,870.41 (82.27)***
		Cycle	120.13 (44.82)***	89.06 (42.76)**	75.11 (32.12)**	25.22 (32.76)*	50.88 (37.25)*	-72.77 (30.62)**	-12.73 (54.90)
		Cycle²	-15.09 (6.16)**	-11.22 (5.87)*	-8.76 (4.41)*	-2.42 (4.50)	-8.44 (5.12)*	9.68 (4.21)**	2.85 (7.54)
Heart attack (With)	Model 1	Intercept	1,003.37 (112.06)***	1,409.65 (115.69)***	1,902.57 (70.93)***	2,845.89 (177.34)***	3,754.81 (174.30)***	4,798.03 (348.59)***	5,890.09 (512.17)***
		Cycle	2.47 (26.79)	-0.41 (27.65)	42.23 (16.95)***	22.93 (42.39)	70.65 (41.66)*	166.59 (83.33)**	187.20 (122.43)*
	Model 2	Intercept	1,075.66 (151.92)***	1,327.27 (154.94)***	1,895.12 (101.17)***	3,053.53 (207.89)***	3,958.91 (204.30)***	5,164.87 (427.24)***	6,084.36 (718.88)***
		Cycle	-69.82 (101.39)	81.96 (103.40)*	49.68 (67.52)*	-184.71 (138.74)*	-133.45 (136.35)*	-200.24 (285.12)	-7.07 (479.76)
		Cycle²	10.33 (13.93)*	-11.77 (14.20)*	-1.06 (9.27)	29.66 (19.06)*	29.16 (18.73)*	52.41 (39.16)*	27.75 (65.90)
Stroke (Without)	Model 1	Intercept	1,222.10 (68.13)***	1,586.62 (58.70)***	2,239.59 (44.63)***	3,204.74 (35.91)***	4,441.88 (48.70)***	5,868.97 (46.19)***	6,882.65 (74.39)***
		Cycle	13.30 (16.29)*	10.77 (14.03)*	13.90 (10.67)*	6.32 (8.58)*	-6.01 (11.64)	-3.42 (11.04)	5.87 (17.78)
	Model 2	Intercept	1,123.89 (69.16)***	1,513.05 (66.33)***	2,181.33 (49.13)***	3,192.43 (50.57)***	4,383.24 (56.29)***	5,938.71 (44.62)***	6,891.74 (106.07)***
		Cycle	111.51 (46.16)**	84.33 (44.27)*	72.16 (32.79)**	18.63 (33.75)	52.62 (37.57)*	-73.16 (29.78)**	-3.22 (70.79)
		Cycle²	-14.03 (6.34)**	-10.51 (6.08)*	-8.32 (4.50)*	-1.76 (4.64)	-8.38 (5.16)*	9.96 (4.09)**	1.30 (9.72)
Stroke (With)	Model 1	Intercept	784.23 (139.21)***	1,202.11 (117.19)***	1,772.51 (112.15)***	2,427.47 (76.83)***	3,339.85 (173.34)***	4,498.48 (213.58)***	4,770.47 (351.33)***
		Cycle	51.40 (33.28)*	28.51 (28.01)*	27.30 (26.81)*	60.44 (18.37)***	61.07 (41.44)*	72.74 (51.06)*	245.88 (83.98)***
	Model 2	Intercept	613.58 (159.35)***	1,001.48 (92.01)***	1,634.07 (127.86)***	2,436.08 (109.57)***	3,631.62 (141.23)***	4,790.05 (227.53)***	5,176.45 (414.41)***

		Cycle	222.05 (106.34)**	229.14 (61.40)***	165.74 (85.33)*	51.83 (73.13)	-230.70 (94.25)**	-218.83 (151.85)*	-160.11 (276.57)
		Cycle²	-24.38 (14.61)*	-28.66 (8.43)***	-19.78 (11.72)*	1.23 (10.04)	41.68 (12.95)***	41.65 (20.86)*	58.00 (37.99)*
Having no condition	Model 1	Intercept	1,248.26 (72.14)***	1,624.62 (66.77)***	2,316.39 (33.16)***	3,314.61 (31.49)***	4,545.03 (50.81)***	5,999.40 (59.68)***	7,066.13 (72.06)***
		Cycle	8.46 (17.24)	8.20 (15.96)	7.70 (7.93)*	-3.76 (7.53)	-14.60 (12.15)*	-7.81 (14.27)	-16.87 (17.22)*
	Model 2	Intercept	1,138.47 (69.00)***	1,531.51 (69.89)***	2,271.70 (35.68)***	3,319.29 (44.86)***	4,475.66 (54.12)***	6,105.19 (42.78)***	7,129.01 (93.12)***
		Cycle	118.25 (46.05)**	101.32 (46.64)**	52.39 (23.81)**	-8.45 (29.94)	54.77 (36.12)*	-113.60 (28.55)***	-79.76 (62.15)*
		Cycle²	-15.68 (6.33)**	-13.30 (6.41)**	-6.38 (3.27)*	0.67 (4.11)	-9.91 (4.96)*	15.11 (3.92)***	8.98 (8.54)*
Having >=1 condition	Model 1	Intercept	1,154.98 (67.59)***	1,492.23 (64.27)***	2,074.17 (64.81)***	2,942.02 (57.91)***	4,156.12 (71.52)***	5,417.55 (90.79)***	6,422.03 (111.11)***
		Cycle	23.89 (16.16)*	17.46 (15.36)*	22.31 (15.49)*	29.99 (13.84)**	16.13 (17.10)*	33.64 (21.70)*	51.80 (26.56)**
	Model 2	Intercept	1,077.17 (79.87)***	1,437.21 (83.40)***	1,985.24 (68.76)***	2,943.77 (82.70)***	4,162.76 (102.05)***	5,440.44 (128.68)***	6,396.58 (157.71)***
		Cycle	101.70 (53.30)*	72.47 (55.66)*	111.24 (45.89)**	28.24 (55.19)	9.48 (68.10)	10.75 (85.88)	77.24 (105.25)*
		Cycle²	-11.12 (7.32)*	-7.86 (7.65)*	-12.70 (6.30)**	0.25 (7.58)	0.95 (9.35)	3.27 (11.80)	-3.64 (14.46)

*P-value < 0.5; **P-value<0.1; ***P-value<0.05.

a. P represents percentiles (P5, P10, P25, P50, P75, P90, and P95).
b. β = beta coefficients, SE= standard error.
c. “With” and “Without” indicates participants’ health conditions.

Codes for visualization:

```
# Figure 1: Temporal and age-related patterns in average sodium intake (mg/day) stratified by sex among NHANES participants (2003–2018)

# Aggregate data: Compute mean sodium intake for each Age × Sex × NHANES Cycle group
Sodium_health_filtered <- Sodium_all %>%
  filter(RIDAGEYR >= 5) %>%
  group_by(RIDAGEYR, RIAGENDR, Year) %>%
  summarise(Avg_Sodium_Intake = mean(DR1TSODI, na.rm = TRUE), .groups = "drop") %>%
  mutate(RIAGENDR = factor(RIAGENDR, levels = c(1, 2), labels = c("Male", "Female")))  # Convert to categorical with labels

# Define custom colors for sex
sex_colors <- c("Male" = "#1F78B4", "Female" = "#FB9A99")

# Panel A: Scatter plot of average sodium intake by age, sex, and NHANES cycle, with LOWESS curves.
### Create a dataset named Sodium_health_filtered with age (RIDAGEYR) as the x-axis and Avg_Sodium_Intake as the y-axis.
### Blue represents males while pale pink represents females. Darker colors represent more recent NHANES cycles.
# Create the scatter plot, with legend
scatter_plot <- ggplot(Sodium_health_filtered, aes(x = RIDAGEYR, y = Avg_Sodium_Intake, color = RIAGENDR)) +
  geom_point(size = 2.5, alpha = 0.7, position = position_jitter(width = 0.2, height = 0)) +  # Scatter points with slight jitter
  geom_vline(xintercept = 18, linetype = "solid", color = "grey50", linewidth = 0.8) +  # vertical line at age 18
  geom_hline(yintercept = 2300, linetype = "solid", color = "darkgreen", linewidth = 0.8) +  # horizontal line at 2300 mg/day
  geom_smooth(method = "loess", se = FALSE, span = 0.5) +  # Fit LOESS curve without confidence interval
  scale_color_manual(values = sex_colors, name = "Sex") +  # Manual color mapping with correct labels
  labs(title = "Scatter Plot of Average Sodium Intake by Age, Sex, and NHANES Cycle",
       x = "Age (years)",
       y = "Average Sodium Intake (mg/day)") +
  scale_x_continuous(breaks = seq(5, max(Sodium_health_filtered$RIDAGEYR), by = 5)) +  # Label every 5 years
  theme_minimal() +
  theme(
    axis.text.x = element_text(angle = 0, hjust = 0.5),
    legend.text = element_text(size = 14),     # Bigger legend text
    legend.title = element_text(size = 16),    # Bigger legend title
    legend.key.size = unit(1.2, "lines")       # Optional: bigger legend keys
  )

# Print the plot
print(scatter_plot)
```

```
# Assign color gradient
# Assign colors based on gender and NHANES cycle (year)
Sodium_health_filtered <- Sodium_health_filtered %>%
  mutate(
    SexYear = paste(RIAGENDR, Year),  # e.g., "Male 2003"
    SexYear = factor(SexYear)         # ensure proper factor ordering
  )

n_years <- length(unique(Sodium_health_filtered$Year))

# Male: Light sky blue → Deep navy
male_colors <- colorRampPalette(c("#D0E1F9", "#08306B"))(8)
# Female: Pale pink → Deep crimson
female_colors <- colorRampPalette(c("#FEE0D2", "#D47D8E"))(8)

# Combine into named vector for scale
legend_colors <- c(
  setNames(male_colors, paste("Male", sort(unique(Sodium_health_filtered$Year)))),
  setNames(female_colors, paste("Female", sort(unique(Sodium_health_filtered$Year))))
)


Sodium_health_filtered <- Sodium_health_filtered %>%
  group_by(RIAGENDR) %>%
  mutate(color = ifelse(RIAGENDR == "Male", male_colors[as.numeric(factor(Year))],
                        female_colors[as.numeric(factor(Year))]))

# Panel B: LOWESS curves fitted separately for each NHANES cycle.
# Create a dataset named Sodium_health_filtered with age (RIDAGEYR) as the x-axis and
Avg_Sodium_Intake as the y-axis.
### Blue represents males while pale pink represents females. Darker colors represent more recent
NHANES cycles.
# Create the LOESS curve plot (Single plot for both genders)
loess_plot <- ggplot(Sodium_health_filtered, aes(x = RIDAGEYR, y = Avg_Sodium_Intake, color = SexYear,
group = interaction(Year, RIAGENDR))) +
  geom_smooth(method = "loess", se = FALSE, span = 0.5, linewidth = 1) +
  geom_vline(xintercept = 18, linetype = "solid", color = "grey50", linewidth = 0.8) +
  geom_hline(yintercept = 2300, linetype = "solid", color = "darkgreen", linewidth = 0.8) +
  scale_color_manual(values = legend_colors, name = "Sex × NHANES Cycle") +
  labs(
    title = "LOESS Curves of Average Sodium Intake by Age, Sex, and NHANES Cycle",
    x = "Age (years)",
    y = "Average Sodium Intake (mg/day)"
  ) +
  scale_x_continuous(breaks = seq(5, max(Sodium_health_filtered$RIDAGEYR), by = 5)) +
  scale_y_continuous(limits = c(2000, 6000), breaks = seq(2000, 6000, by = 1000)) +  # <-- New line here
  theme_minimal() +
```

```r
  theme(
    axis.text.x = element_text(angle = 0, hjust = 0.5),
    legend.text = element_text(size = 12),
    legend.title = element_text(size = 14),
    legend.key.height = unit(0.5, "cm"),
    legend.key.width = unit(1.5, "cm")
  )


#Figure 2: Distributions of Sodium Intake Among U.S. Adults (2003–2018)
# Filter to adults only
Densityplot_data <- Sodium_all %>%
  filter(RIDAGEYR >= 18, !is.na(DR1TSODI) & DR1TSODI >= 0)

# Create Year × Sex label
Densityplot_data <- Densityplot_data %>%
  mutate(
    Year = as.character(Year),  # ensure Year is character
    RIAGENDR = recode(as.character(RIAGENDR), "1" = "Male", "2" = "Female"),  # recode if needed
    YearSex = paste(Year, RIAGENDR),  # Now this should work
    YearSex = factor(YearSex)
  )

# Define color palettes
years <- sort(unique(Densityplot_data$Year))
n_years <- length(years)

# Male: Light blue → Navy
blue_palette <- colorRampPalette(c("#D0E1F9", "#08306B"))(n_years)
# Female: Light pink → Deep rose
red_palette  <- colorRampPalette(c("#FEE0D2", "#D47D8E"))(n_years)

# Create combined palette
palette_labels <- c(paste(years, "Male"), paste(years, "Female"))
combined_palette <- setNames(c(blue_palette, red_palette), palette_labels)

# Re-factor to match palette
Densityplot_data$YearSex <- factor(Densityplot_data$YearSex, levels = palette_labels)

### Panel A: Cumulative distribution of sodium intake (mg/day), stratified by sex.
### Create a dataset named Densityplot_data with day 1 sodium intake (mg/day) (DR1TSODI) as the x-
axis.
### Blue represents males while pale pink represets females. Darker colors represent more recent
NHANES cycles.
ggplot(Densityplot_data, aes(x = DR1TSODI, color = YearSex)) +
  stat_ecdf(geom = "step", aes(group = YearSex), linewidth = 0.5) +
  geom_vline(xintercept = 2300, color = "green4", linetype = "solid", linewidth = 0.9) +
  labs(
```

```
    title = "Cumulative Distribution of Sodium Intake Among Adults by Sex and NHANES Cycle",
    x = "Sodium Intake (mg/day)",
    y = "Cumulative Proportion"
  ) +
  scale_color_manual(values = combined_palette, name = "Cycle × Sex") +
  scale_x_continuous(labels = comma) +
  scale_y_continuous(labels = percent_format(accuracy = 1)) +
  coord_cartesian(xlim = c(0, 20000)) +
  theme_minimal() +
  theme(
    legend.position = "bottom",
    axis.title = element_text(size = 14),
    axis.text = element_text(size = 14),
    legend.text = element_text(size = 13),
    legend.title = element_text(size = 14)
  )
```

### Panel B: Zoomed-in view of Cumulative distribution of sodium intake between 1,000 and 6,000 mg/day, stratified by sex.

```
### Create a dataset named Densityplot_data with day 1 sodium intake (mg/day) (DR1TSODI) as the x-axis.
### Blue represents males while pale pink represents females. Darker colors represent more recent NHANES cycles.
ggplot(Densityplot_data, aes(x = DR1TSODI, color = YearSex)) +
  stat_ecdf(geom = "step", aes(group = YearSex), linewidth = 0.7) +
  geom_vline(xintercept = 2300, color = "green4", linetype = "solid", linewidth = 0.9) +
  labs(
    title = "Cumulative Distribution of Sodium Intake Among Adults by Sex and NHANES Cycle",
    x = "Sodium Intake (mg/day)",
    y = "Cumulative Proportion"
  ) +
  scale_color_manual(values = combined_palette, name = "Cycle × Sex") +
  scale_x_continuous(labels = comma) +
  scale_y_continuous(labels = percent_format(accuracy = 1)) +
  coord_cartesian(xlim = c(1000, 6000)) +  # Zoom in here!
  theme_minimal() +
  theme(
    legend.position = "bottom",
    axis.title = element_text(size = 14),
    axis.text = element_text(size = 14),
    legend.text = element_text(size = 13),
    legend.title = element_text(size = 14)
  )

# Calculate percentiles
percentiles <- quantile(Densityplot_data$DR1TSODI, probs = c(0.05, 0.10, 0.25, 0.5, 0.75, 0.9, 0.95), na.rm = TRUE)
```

### Panel C: Kernel density plot of sodium intake (mg/day), stratified by sex.

```
### Create a dataset named Densityplot_data with day 1 sodium intake (mg/day) (DR1TSODI) as the x-
axis.
### Blue represents males while pale pink represents females.
ggplot(Densityplot_data, aes(x = DR1TSODI, fill = RIAGENDR)) +
  # Add percentile background shading
  annotate("rect", xmin = 0, xmax = percentiles[1], ymin = -Inf, ymax = Inf, fill = "#006400", alpha = 0.3) +
# P0–P5
  annotate("rect", xmin = percentiles[1], xmax = percentiles[2], ymin = -Inf, ymax = Inf, fill = "#228B22",
alpha = 0.15) +  # P5–P10
  annotate("rect", xmin = percentiles[2], xmax = percentiles[3], ymin = -Inf, ymax = Inf, fill = "#90EE90",
alpha = 0.15) +  # P10–P25
  annotate("rect", xmin = percentiles[3], xmax = percentiles[4], ymin = -Inf, ymax = Inf, fill = "#FFE4E1",
alpha = 0.15) +  # P25–P50
  annotate("rect", xmin = percentiles[4], xmax = percentiles[5], ymin = -Inf, ymax = Inf, fill = "#FA8072",
alpha = 0.15) +  # P50–P75
  annotate("rect", xmin = percentiles[5], xmax = percentiles[6], ymin = -Inf, ymax = Inf, fill = "#CD5C5C",
alpha = 0.15) +  # P75–P90
  annotate("rect", xmin = percentiles[6], xmax = percentiles[7], ymin = -Inf, ymax = Inf, fill = "#8B0000",
alpha = 0.15) +  # P90–P95
  annotate("rect", xmin = percentiles[7], xmax = 20000, ymin = -Inf, ymax = Inf, fill = "#8B0001", alpha =
0.2) +  # P90–P95


  # Add density
  geom_density(alpha = 0.6, color = NA) +

  # Labels and styling
  labs(
    title = "Density Plot of Sodium Intake by Sex with Shaded Percentile Regions",
    x = "Sodium Intake (mg/day)",
    y = "Density"
  ) +
  scale_fill_manual(values = c("Male" = "#08306B", "Female" = "#D47D8E")) +
  coord_cartesian(xlim = c(0, 20000))+
  scale_x_continuous(
    breaks = seq(0, 20000, by = 2000),
    labels = comma
  )+
  theme_minimal()+
  theme(
    axis.title = element_blank(),     # Removes axis titles
    legend.text = element_text(size = 14),     # Bigger legend text
    legend.position = "none",        # Removes legend
    plot.title = element_blank()
  )
```
